# Short-Stay Units vs Routine Admission From the Emergency Department in Patients With Acute Heart Failure: The SSU-AHF Randomized Clinical Trial

**DOI:** 10.1001/jamanetworkopen.2023.50511

**Published:** 2024-01-10

**Authors:** Peter S. Pang, David A. Berger, Simon A. Mahler, Xiaochun Li, Susan J. Pressler, Kathleen A. Lane, Jason J. Bischof, Douglas Char, Deborah Diercks, Alan E. Jones, Erik P. Hess, Phillip Levy, Joseph B. Miller, Arvind Venkat, Nicholas E. Harrison, Sean P. Collins

**Affiliations:** 1 Department of Emergency Medicine, Indiana University School of Medicine, Indianapolis; 2 Department of Emergency Medicine, William Beaumont Hospital, Royal Oak, Michigan; 3 Wake Forest University School of Medicine, Winston-Salem, North Carolina; 4 Department of Biostatistics and Health Data Science, Indiana University School of Medicine, Indianapolis,; 5 Indiana University School of Nursing, Indianapolis; 6 Department of Emergency Medicine, The Ohio State University, Columbus; 7 Department of Emergency Medicine, Washington University in St Louis, St Louis, Missouri; 8 Department of Emergency Medicine, University of Texas Southwestern Medical Center, Dallas; 9 Department of Emergency Medicine, University of Mississippi Medical Center, Jackson; 10 Department of Emergency Medicine, Vanderbilt University School of Medicine, Nashville, Tennessee; 11 Wayne State University School of Medicine and Integrative Biosciences Center, Detroit, Michigan; 12 Department of Emergency Medicine, Henry Ford Hospital, Detroit, Michigan; 13 Department of Emergency Medicine, Allegheny Health Network, Pittsburgh, Pennsylvania; 14 Department of Emergency Medicine, Vanderbilt University School of Medicine and Veterans Affairs Tennessee Valley Healthcare System, Geriatric Research, Education and Clinical Center, Nashville, Tennessee

## Abstract

**Importance:**

More than 80% of patients who present to the emergency department (ED) with acute heart failure (AHF) are hospitalized. With more than 1 million annual hospitalizations for AHF in the US, safe and effective alternatives are needed. Care for AHF in short-stay units (SSUs) may be safe and more efficient than hospitalization, especially for lower-risk patients, but randomized clinical trial data are lacking.

**Objective:**

To compare the effectiveness of SSU care vs hospitalization in lower-risk patients with AHF.

**Design, Setting, and Participants:**

This multicenter randomized clinical trial randomly assigned low-risk patients with AHF 1:1 to SSU or hospital admission from the ED. Patients received follow-up at 30 and 90 days post discharge. The study began December 6, 2017, and was completed on July 22, 2021. The data were analyzed between March 27, 2020, and November 11, 2023.

**Intervention:**

Randomized post-ED disposition to less than 24 hours of SSU care vs hospitalization.

**Main Outcomes and Measures:**

The study was designed to detect at least 1-day superiority for a primary outcome of days alive and out of hospital (DAOOH) at 30-day follow-up for 534 participants, with an allowance of 10% participant attrition. Due to the COVID-19 pandemic, enrollment was truncated at 194 participants. Before unmasking, the primary outcome was changed from DAOOH to an outcome with adequate statistical power: quality of life as measured by the 12-item Kansas City Cardiomyopathy Questionnaire (KCCQ-12). The KCCQ-12 scores range from 0 to 100, with higher scores indicating better quality of life.

**Results:**

Of the 193 patients enrolled (1 was found ineligible after randomization), the mean (SD) age was 64.8 (14.8) years, 79 (40.9%) were women, and 114 (59.1%) were men. Baseline characteristics were balanced between arms. The mean (SD) KCCQ-12 summary score between the SSU and hospitalization arms at 30 days was 51.3 (25.7) vs 45.8 (23.8) points, respectively (*P* = .19). Participants in the SSU arm had 1.6 more DAOOH at 30-day follow-up than those in the hospitalization arm (median [IQR], 26.9 [24.4-28.8] vs 25.4 [22.0-27.7] days; *P* = .02). Adverse events were uncommon and similar in both arms.

**Conclusions and Relevance:**

The findings show that the SSU strategy was no different than hospitalization with regard to KCCQ-12 score, superior for more DAOOH, and safe for lower-risk patients with AHF. These findings of lower health care utilization with the SSU strategy need to be definitively tested in an adequately powered study.

**Trial Registration:**

ClinicalTrials.gov Identifier: NCT03302910

## Key Points

**Question:**

Instead of hospital admission from the emergency department, does a short-stay unit (SSU) strategy of care improve quality of life for lower-risk patients with acute heart failure?

**Findings:**

In this randomized clinical trial of 193 patients, there were no significant differences in quality of life at 30-day follow-up and no safety differences, although there were more days alive and out of hospital in the SSU arm. Outcomes were underpowered due to low enrollment during the COVID-19 pandemic.

**Meaning:**

These findings show that for lower-risk patients with acute heart failure, SSUs may be a reasonable alternative to hospitalization.

## Teaser

This randomized clinical trial compares the effectiveness of care delivered through a short-stay unit (SSU) vs hospitalization for low-risk patients with acute heart failure (AHF).

## Introduction

Health care utilization for acute heart failure (AHF) is an important patient and health services burden. In the US, approximately 6 million people have heart failure,^1^ with mortality rates increasing since 2012 and hospitalization rates increasing in the past decade.^2^ The cost of heart failure is projected to increase from $3.7 billion in 2012 to $69.8 billion in 2030, with hospitalization for AHF being the greatest contributor.^1,3^ Hospitalization is also a marker for serious adverse events. Compared with patients with chronic heart failure not hospitalized, the 1-year all-cause mortality rate is nearly 4 times higher for those who are hospitalized.^4^ Patients of lower socioeconomic status are disproportionately affected by both hospitalization and rehospitalization.^5^

The emergency department (ED) accounts for 70% to 80% of all AHF admissions.^6,7,8^ Patients initially diagnosed and treated for AHF in the ED are admitted to the hospital 80% to 90% of the time, yet as many as one-half of these admissions may be avoidable.^6,7,8^ These potentially avoidable admissions are characterized by patients at lower risk for adverse events, such as those with higher blood pressure and lower natriuretic peptide levels.^8,9^ Hospitalizing or rehospitalizing such patients may unnecessarily expose them to in-hospital adverse events and reduced quality of life.^10^ Furthermore, approximately 25% of discharged patients with AHF are rehospitalized within 30 days. Safe alternatives to hospitalization are needed.^7,11,12^

Brief observation (<24 hours), often referred to as short-stay unit (SSU) management, has been supported for lower-risk patients with AHF by observational studies and consensus statements.^13,14,15^ Prior work has shown a shorter length of stay in SSU patients compared with risk-matched inpatients (25.7 vs 58.5 hours),^13,14^ but past studies were limited by their nonrandomized design. Thus, we designed the Using Short Stay Units Instead of Routine Admission to Improve Patient Centered Health Outcomes for Acute Heart Failure Patients (SSU-AHF) trial to overcome prior limitations and evaluate outcomes in the setting of a randomized trial.

## Methods

### Study Design

The SSU-AHF trial was a multicenter, randomized, comparative effectiveness study comparing 2 strategies of care for ED patients with AHF: SSU treatment vs hospitalization. The study was conducted between December 6, 2017, and July 22, 2021.

The SSU nomenclature was informed based on feedback from patient focus groups.^16,17^ To address the variability in SSU treatment across hospitals, we defined the intervention arm as observation or SSUs where the length-of-stay goal was less than 24 hours and patients were treated by ED clinicians. More in-depth details regarding the study design have been previously published.^18^ The COVID-19 pandemic required significant changes to the study design, including the primary outcome and overall sample size. While patients were enrolled from the ED throughout the trial, during the pandemic, clinical research in the ED and use of the observation unit for patients with shortness of breath were either completely abandoned or used for different types of patients with COVID-19. When the trial resumed during the pandemic, clinical and research staffing challenges required reinitiation of start-up activities. As the pandemic waxed and waned, such activities occurred multiple times. When it became clear that enrollment projections would not reach prior goals used to inform sample size calculations, the study leadership, in consultation with the funding agency, made changes in the primary outcome based on a new enrollment projection. The original trial protocol is provided in Supplement 1.

All sites first obtained institutional review board approval prior to study enrollment. All patients provided written informed consent before randomization, and the study was registered with ClinicalTrials.gov (NCT03302910). This study followed the CONSORT reporting guideline.^19^

The Indiana University School of Medicine acted as the study coordinating center responsible for all study operations and data operations. An independent data safety monitoring board was created to ensure the safety of all trial participants. Adverse events were collected for the first 5 days after randomization. Each reported adverse event was described by its duration (ie, start and end dates); regulatory seriousness criteria, if applicable; and suspected relationship to the study protocol in accordance with definitions set forth by each institutional review board. In general, these relationships were categorized as likely, possible, unlikely, and not related. Adverse events were assessed via medical record review and during patient follow-up.

### Study Population and Setting

The target population was lower-risk patients who presented to the ED with signs and symptoms of AHF. Final eligibility criteria are listed in eTable 1 in Supplement 2 and were modified from the original study protocol. Importantly, only patients who the ED clinician planned to hospitalize were eligible for enrollment. Patients were enrolled at 12 academic hospital sites in the following states: Indiana, Tennessee, Michigan, North Carolina, Pennsylvania, Ohio, Mississippi, Alabama, and Missouri (eTable 2 in Supplement 2). One site did not enroll any patients, as study start-up occurred proximate to the pandemic.

### Randomization and Study Procedures

Race and ethnicity were obtained by the study team via self-report directly from the participants during the consent and randomization process. Eligible patients were randomized 1:1 to either hospital admission or SSU treatment through permuted block randomization (block sizes of 2, 4, and 6). Follow-up data were collected via telephone at 30 (±7) and 90 (±30) days post discharge from either the hospital or SSU (up to 120 total days after randomization per protocol). There were no further in-person visits required once discharged. Follow-up calls assessed vital status, all-cause rehospitalizations, ED revisits (via patient recall and electronic health record [EHR] search), and quality of life using the 12-item Kansas City Cardiomyopathy Questionnaire (KCCQ-12) short form. All decisions related to clinical testing and therapy were at the discretion of the clinical teams and were not dictated by study protocol.^18^

All SSU patients were monitored by telemetry. Four principles of SSU AHF management were outlined in the SSU management protocol^18^: (1) relief of heart failure symptoms and signs, (2) decongestion and correction of any electrolyte imbalances, (3) hemodynamic improvement, and (4) robust care transitions with an emphasis on guideline-directed medication reconciliation and guideline-recommended therapy at discharge.^15,20,21^ Our management strategies were adapted from European Society of Cardiology and Society of Chest Pain Centers guidelines.^21^ We augmented these guidelines by providing greater detail regarding vasodilator and diuretic dosing. Vasodilators were suggested to be considered when congestion persisted and systolic blood pressure was greater than 135 mm Hg. The SSU discharge criteria were taken from the Society of Chest Pain Centers guidelines on observation unit care.^15^ Patients who had an acute event during their SSU stay or were not ready for discharge after the SSU management phase were hospitalized.

Hospitalization for AHF was chosen as the comparator. Acute heart failure treatment was left to the discretion of the inpatient care teams. In keeping with the pragmatic study design, each study arm used existing transitional care programs to assist with the postdischarge period.

### Study Outcomes

The original primary outcome was days alive and out of hospital (DAOOH) at 30 days. Original study outcomes are listed in eTable 3 in Supplement 2. Because of the COVID-19 pandemic, we revised the enrollment goals and changed our focus to 1 of our secondary outcomes, quality of life, as measured by the KCCQ-12 short form.^22^ We anticipated adequate power based on prior data from other studies to change our primary focus to KCCQ-12 scores (eTable 4 in Supplement 2). The KCCQ-12 has been used in prior AHF studies and is sensitive to the patient’s changing clinical condition, and a change of 5 points or more has been accepted as a clinically important difference.^23,24^ Prior work by our study group suggested that changes in KCCQ-12 scores are associated with rehospitalization.^23^ Thus, we hypothesized that SSU patients would have a greater quality of life due to fewer hospital days. Specifically, we hypothesized that the SSU strategy of care compared with hospitalization would lead to a significant improvement (≥5 points) in quality of life scores at 30 days post discharge. After this change in our primary outcome, the original 30-day DAOOH became a secondary outcome. Other outcomes, such as caregiver burden assessments, were eliminated because families were not allowed to accompany patients in the hospital.

### Statistical Analysis

Data analyses were performed between March 27, 2020, and November 11, 2023. In accordance with the intention-to-treat principle, participants were analyzed by the arm to which they were randomized (statistical analysis plan provided in Supplement 1). Patient baseline characteristics are presented using means (SDs) or medians (IQRs) for continuous variables or frequency and percentages as appropriate. Group comparisons of continuous variables were based on either 2-sample *t* tests (under normality) or Wilcoxon rank sum test. Categorical data comparisons are presented based on Fisher exact test.

#### KCCQ-12 Quality-of-Life Analysis

The KCCQ-12 mean score was compared between the 2 arms using a 2-sample *t* test. We analyzed the KCCQ-12 results 3 ways. First, patients who died without a KCCQ-12 score were excluded. Second, we set the KCCQ-12 to 0 for those who died without the 30-day KCCQ-12 score and include these participants in the analysis. Third, we created a composite binary end point of KCCQ-12 score less than *c* or death, where *c* is a threshold. We used both the 25th percentile and median for this threshold. We used *t* and Fisher exact tests to compare these outcomes. The 3 analysis schemes allowed us to understand how robust the comparison of KCCQ-12 scores is with respect to different thresholds of KCCQ-12 scores when death occurred.

To provide context for the responder analysis, we have included eTable 5 in Supplement 2 to summarize the baseline characteristics of KCCQ-12 responders in each treatment arm in order to facilitate assessment of the balance of the treatment groups among responders. The KCCQ-12 change scores were compared between the 2 arms using a 2-sample *t* test.

#### Secondary Analysis: DAOOH Outcome

The EHR time stamps were used to capture time of ED arrival, time leaving the ED, time of arrival to SSU or inpatient admission, and time of discharge. Any time spent in the ED, SSU, or inpatient admission was counted against the participant’s DAOOH. If length of stay in the SSU was 29 hours, this time counted as 1.2 days. The DAOOH outcome includes time from randomization, capturing the index ED or hospital visit. Additional analyses are reported from time of discharge. The DAOOH was compared between the 2 treatment groups using a Wilcoxon rank sum test.

A composite outcome of all-cause mortality and rehospitalization was defined as the time from randomization to either all-cause death or rehospitalization at 30 and 90 days. The 2 treatments groups were compared using a log-rank test. To account for the influence of differences in site management, a stratified log-rank test was used. The Fisher exact test was used to compare the proportions of participants who had either all-cause mortality or rehospitalization within 30 and 90 days from randomization.

A 2-sided *P* < .05 was considered statistically significant without adjustment for multiple comparisons. Statistical tables and listings and analyses were produced using SAS, version 9.4 software (SAS Institute, Inc).

## Results

### Participants

**Figure 1.**
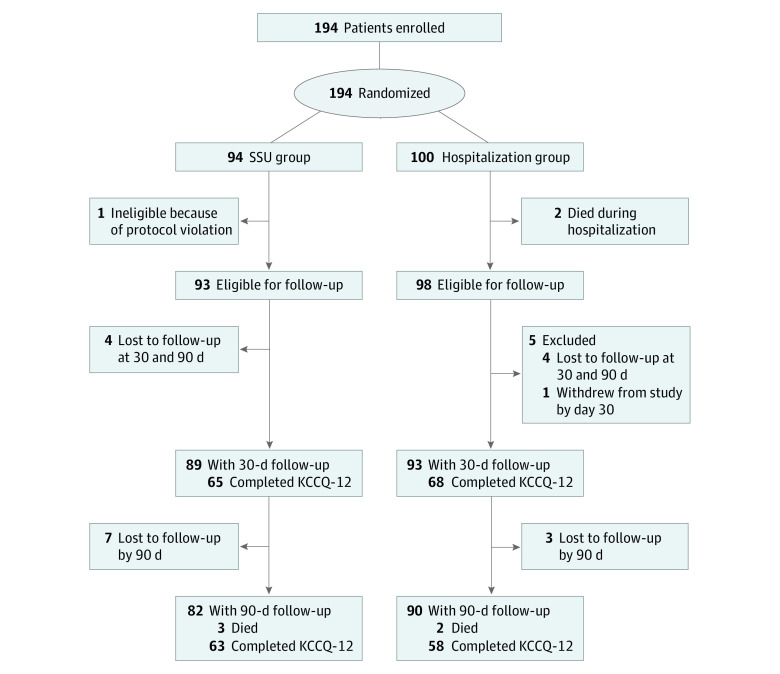
CONSORT Diagram KCCQ-12 indicates 12-item Kansas City Cardiomyopathy Questionnaire; SSU, short-stay unit.

Starting December 6, 2017, through final follow-up on August 5, 2021, 194 patients were enrolled across 12 sites: 94 in the SSU arm and 100 in the hospitalization arm (with 1 participant found ineligible after randomization due to a protocol violation in the SSU arm) (Figure 1). Two patients died during hospitalization in the hospitalization arm. Of the 193 patients enrolled, the mean (SD) age was 64.8 (14.8) years; 79 (40.9%) were women and 114 (59.1%) men; of 192 with available data, 108 (56.3%) self-identified as Black, 2 (1.0%) as Hispanic or Latino, 80 (41.7%) as White, and 4 (2.1%) as other race (including 1 each of American Indian or Alaska Native, Arabic or Middle Eastern, Asian Indian, and Filipino). At baseline, characteristics between arms were similar (Table 1). Participants had a mean (SD) left ventricular ejection fraction (LVEF) of 36.8% (16.5%) in the SSU arm and 41.4% (15.3%) in the hospitalization arm (*P* = .12). There were numerically more patients with a reduced LVEF in the SSU arm. The treatment groups were similar in terms of baseline vital signs and laboratory values. There were no observed differences between the SSU and hospitalization arms with respect to the proportion of study patients receiving furosemide in the ED (87 [93.5%] vs 87 [87.0%]; *P* = .15) or the total intravenous furosemide dose while in the ED (61.0 [30.4] vs 55.5 [32.0] mg; *P* = .26). There were also no differences in other initial ED therapy (eTable 6 in Supplement 2). Regarding medications given on the day of discharge, hospitalized participants were more likely to receive anticoagulation (eTable 7 in Supplement 2).

**Table 1.** Baseline Characteristics

Variable	Short-stay unit		Hospitalization	
	No. of patients	No. (%)	No. of patients	No. (%)
Age, mean (SD), y	93	63.4 (14.3)	100	66.1 (15.3)
Age ≥75 y	93	21 (22.6)	100	30 (3.0)
Sex				
Male	93	61 (65.6)	100	53 (53.0)
Female	93	32 (34.4)	100	47 (47.0)
Race				
Black	92	51 (55.4)	100	57 (57.0)
White	92	38 (41.3)	100	42 (42.0)
Other^a^	92	3 (3.3)	100	1 (1.0)
Ethnicity				
Hispanic or Latino	92	2 (2.2)	98	0
Any ED visit or hospitalization in the past 6 mo	93	61 (65.6)	100	71 (71.0)
Coronary artery disease	93	49 (52.7)	100	50 (5.0)
Hypertension	93	82 (88.2)	100	90 (9.0)
Diabetes	93	52 (55.9)	100	51 (51.0)
Chronic kidney disease	93	34 (36.6)	100	42 (42.0)
Depression history	93	23 (24.7)	100	26 (26.0)
Asthma or COPD	93	31 (33.3)	100	34 (34.0)
Pacemaker	93	22 (23.7)	100	16 (16.0)
ICD	93	21 (22.6)	99	25 (25.3)
Atrial fibrillation or atrial flutter	93	37 (39.8)	100	48 (48.0)
CVA or TIA	93	18 (19.4)	100	22 (22.0)
Heart rate, mean (SD), beats/min	93	82.5 (15.0)	99	83.7 (16.8)
Respiratory rate, mean (SD), breaths/min	93	2.4 (4.9)	100	19.4 (2.7)
Blood pressure, mean (SD), mmHg				
Systolic	93	142.3(23.5)	100	142.5 (28.7)
Diastolic	93	84.1 (2.1)	100	83.7 (2.8)
Oxygen saturation, mean (SD), %	93	97.2 (2.4)	100	97.1 (2.3)
BMI, mean (SD)	32	37.4 (11.8)	36	35.6 (11.3)
Sodium, median (IQR), mEq/L	93	139 (137-141)	100	139 (137-141)
Creatinine, median (IQR), mg/dL	93	1.15 (0.93-1.43)	100	1.25 (0.98-1.56)
BNP, median (IQR), pg/mL	89	647 (265-1445)	94	646.5 (326-1488)
Troponin I, median (IQR), ng/L	89	0.03 (0.02-0.05)	90	0.03 (0.02-0.06)
LVEF, %				
Mean (SD)	54	36.8 (16.5)	63	41.4 (15.3)
<40%	54	34 (63.0)	63	27 (42.9)
40%-55%	54	110 (2.4)	63	25 (39.7)
>55%	54	9 (16.7)	63	11 (17.5)

Abbreviations: BMI, body mass index (measured by weight in kilograms divided by height in meters squared); BNP, brain-type natriuretic peptide; COPD, chronic obstructive pulmonary disease; CVA, cerebrovascular accident; ED, emergency department; ICD, implantable cardioverter-defibrillator; LVEF, left ventricular ejection fraction; TIA, transient ischemic attack.

^a^ Other included 1 each identifying as American Indian or Alaska Native, Arabic or Middle Eastern, Asian Indian, and Filipino.

### KCCQ-12 Quality-of-Life Outcome

**Figure 2.**
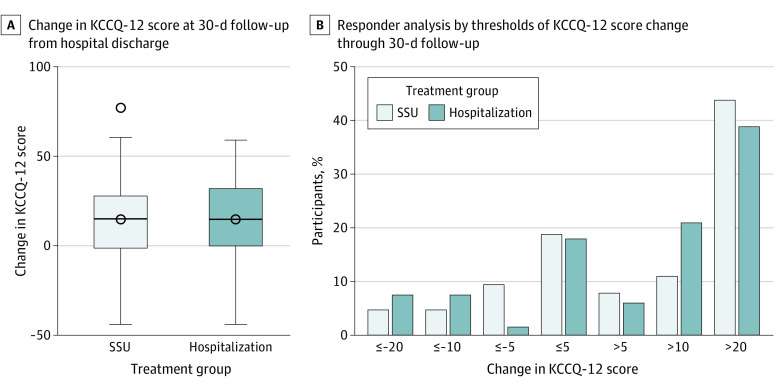
Change in 12-Item Kansas City Cardiomyopathy Questionnaire (KCCQ-12) Scores at 30 Days Boxes indicate the IQRs, with the top and bottom boundaries indicating the upper and lower quartile, respectively. The center line is the median, and the whiskers extend to the minimum and maximum values. SSU indicates short-stay unit.

Of the 193 participants randomized, 4 (4.3%) in the SSU arm and 4 (4.0%) in the hospitalization arm were lost to follow-up at 30 days, and 1 from the hospitalization arm withdrew from the study on day 3. At the time of SSU or hospital discharge, 91 participants (97.9%) in the SSU arm and 97 (97.0%) in the hospitalization arm completed the KCCQ-12, with mean (SD) scores of 35.5 (22.2) and 33.1 (23.1), respectively (*P* = .47). Table 2 shows the breakdown of the KCCQ-12 components at baseline (time of SSU or hospital discharge) and 30 days. In both arms (SSU, 64 participants; hospitalization, 67 participants), KCCQ-12 scores at day 30 improved from discharge to well above the 5 points deemed clinically significant.^23,24^ At day 30, there was no significant difference in KCCQ-12 scores (SSU mean [SD] score, 51.3 [25.7] points [n = 65]; hospitalization mean [SD] score, 45.8 [23.8] [n = 68]; mean difference, 5.6 points; *P* = .19) (Figure 2A). When a composite binary end point of KCCQ-12 was created with KCCQ-12 less than *c* or death, where *c* is a threshold (we used both 25th percentile and median or equals 0 for deaths), there were also no significant differences. Figure 2B shows the analysis of KCCQ-12 responders by established thresholds of clinically important differences in KCCQ-12 scores.^24^ eTable 5 in Supplement 2 shows that the baseline characteristics of the KCCQ-12 responders were comparable between the treatment groups, with the exception of the categorized LVEF.

**Table 2.** Primary and Secondary Outcomes: 12-Item Kansas City Cardiomyopathy Questionnaire (KCCQ-12) at Baseline (Discharge) and 30-Day Follow-Up

Outcomes	Short-stay unit		Hospitalization		*P* value
	No. of patients	Mean (SD) or No. (%)	No. of patients	Mean (SD) or No. (%)	
**Primary**					
Discharge KCCQ-12 score	91	35.5 (22.2)	97	33.1 (23.1)	.47
Discharge KCCQ-12 score for patients with a 30-d score	64	36.0 (2.6)	67	3.9 (21.7)	.17
30-d KCCQ-12 score	65	51.3 (25.7)	68	45.8 (23.8)	.19
30-d KCCQ-12 score for patients with both discharge and 30-d score	64	51.0 (25.7)	67	45.8 (23.9)	.23
Change in KCCQ-12 score at 30 d from discharge	64	15.0 (23.3)	67	15.0 (23.2)	.99
**Secondary**					
30-d Follow-up	93	89 (95.7)	98	93 (94.9)	>.99
SF-12 physical component scale score	63	37.6 (8.4)	67	36.4 (8.1)	.43
SF-12 mental component scale score	63	4.1 (7.0)	67	38.4 (7.1)	.18
KCCQ-12 score, mean (SD)	65	51.3 (25.7)	68	45.8 (23.8)	.19
30-d DAOOH from randomization, median (IQR)	89	26.9 (24.4-28.8)	95^a^	25.4 (22.0-27.7)	.02
30-d DAOOH based on original hospital discharge, median (IQR)	89	3.0 (27.0-3.0)	93	3.0 (27.4-3.0)	.23
30-d All-cause death or rehospitalization^b^	84	16 (19)	93	18 (19.4)	.94^c^
Unscheduled ED visit during 30 d post randomization	89	24 (27.0)	93	17 (18.3)	.21
Unscheduled ED visit for heart failure during 30 d post randomization	89	15 (16.9)	93	18 (19.4)	.28
Unscheduled hospital admission during 30 d post randomization	89	16 (18.0)	93	17 (18.3)	>.99
Unscheduled hospital admission for heart failure during 30 d post randomization	89	12 (13.5)	93	10 (1.8)	.65

Abbreviations: DAOOH, days alive and out of hospital; ED, emergency department; SF-12, 12-Item Short Form.

^a^ Includes 2 patients who died during hospitalization.

^b^ Only for participants with follow-up data.

^c^ Log-rank *P* value tested time from randomization to the earlier of all-cause death or rehospitalization (in days).

### Secondary Outcome: DAOOH at 30 Days

**Figure 3.**
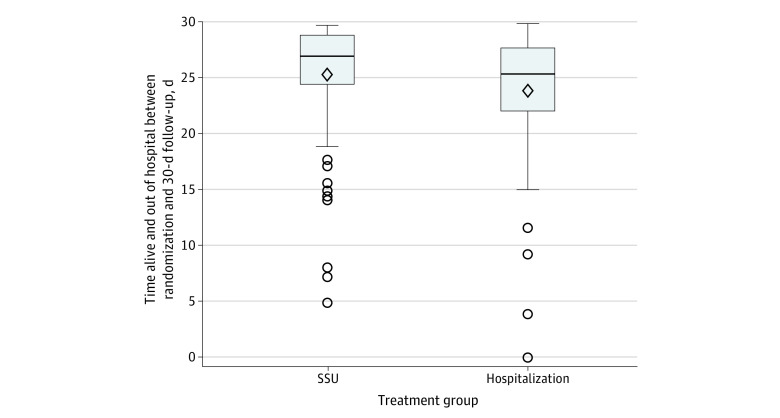
Days Alive and Out of the Hospital Between Randomization and 30-Day Follow-Up Boxes indicate the IQRs, with the top and bottom boundaries indicating the upper and lower quartile, respectively. The center line is the median, and the whiskers extend to the minimum and maximum values. The diamond represents the mean, and circles represent outliers. SSU indicates short-stay unit.

No additional deaths occurred in either arm after hospital discharge through 30 days. Participants in the SSU arm had a significant 1.6 more DAOOH (median, 26.9 days; IQR, 24.4-28.8 days) vs the hospitalization arm (median, 25.4 days; IQR, 22.0-27.7 days; *P* = .02) (Figure 3). There were no differences between arms for either 30- or 90-day all-cause death or rehospitalization. Table 2 shows the original primary outcome as well as other secondary outcomes of interest. eTable 8 in Supplement 2 shows 90-day secondary outcomes.

### Other Outcomes

Of the participants randomized to the SSU arm, 39 (41.9%) required hospitalization after SSU treatment (eTable 9 in Supplement 2). None of the patients in the hospitalization arm crossed over to the SSU strategy. For participants who required hospitalization from the SSU (SSU treatment failure), their number of hospital days, including the ED stay, was greater than for those randomized to the hospitalization arm (median, 4.0 [IQR, 2.8-10.1] vs 3.1 [IQR 2.0-5.9] days; *P* = .03). When followed through 30 days, however, there was no significant difference in the number of days in the hospital and ED when counting the initial hospital stay (mean [SD], 7.6 [6.5] vs 5.6 [4.8] SSU vs hospitalization, respectively; *P* = .09).

A total of 7 deaths occurred in the study by 90 days, 3 in the SSU arm and 4 in the hospitalization arm (Figure 1), with only 1 death occurring within the adverse event monitoring period. In the SSU arm, 15 participants (16.1%) experienced an adverse event vs 16 (16.0%) in the hospitalization arm.

## Discussion

In this multicenter, randomized clinical trial, no differences between SSU treatment and hospitalization were seen in KCCQ-12 scores at day 3. However, there was a significant increase in the number of DAOOH at 30 days in the SSU arm. While both treatment strategies resulted in clinically important changes in KCCQ-12 scores from baseline, lower-than-expected enrollment during the COVID-19 pandemic left the study underpowered to detect a significant difference between the strategies. There were no differences in adverse events between groups. Our findings build on past work where SSU as an alternative to hospitalization from the ED appeared to be a safe option in lower-risk patients with AHF seen in the ED.^6,25,26,27^ Prior to this study, only observational data combined with expert consensus supported SSU management of AHF.^13,14,15,28,29^ Given that 41.9% of SSU participants required hospitalization and had a longer combined ED and hospital length of stay than those randomized to hospitalization, the significant difference in 30-day DAOOH favoring SSU is notable. Additionally, the high number of SSU treatment failures suggests that further refinement of SSU eligibility criteria, SSU management procedures, or both may strengthen the SSU strategy.

Currently, more than 80% of patients who present to the ED with AHF are hospitalized.^7^ The primary concern for SSU management of AHF has been safety. Patients with heart failure tend to be older with multiple comorbidities and polypharmacy. While a subset of patients with AHF can be quantified as lower risk, very few are truly at low risk of 30-day events.^8,11,25^ Our trial adds to the prior literature by suggesting that SSUs offer an alternative setting for trying novel approaches to risk stratification, including ongoing treatment, and assessment for rigorous self-care.^11,27,30^ Collins et al^27^ recently suggested that direct discharge from the ED in this low-risk population is feasible and generally safe. Additionally, Lee et al^26^ recently used a structured clinical decision instrument to identify low-risk patients with AHF to facilitate earlier ED and hospital discharge to lower their DAOOH. Our finding of differences in total number of hospital days through day 30 is hypothesis generating and suggests that subsequent strategy trial designs should consider this as a key efficacy outcome. Given current high admission rates, SSUs may be a reasonable first step prior to emergency physicians discharging patients directly from the ED. Continued development and implementation of risk-stratification tools may facilitate early, safe discharge in ED patients with AHF.

Quality of life is important to patients and is an established patient-reported outcome. While our study did not show a statistically significant benefit of SSU over hospitalization, the fact that hospitalization did not improve quality of life over SSU supports the consideration of SSU management instead of routine hospital admission. Some patients may prefer a single hospitalization vs ED discharge and a return visit, which might be inferred from the lack of difference in quality of life scores; however, the chance to go home and potentially stay home, especially for lower-risk patients, may also be important to patients. Our study group’s preliminary work asking for patient input into our study design supports this concept of home care.^18,27^

### Limitations

This study has some limitations. The relatively small sample size mitigates any robust conclusions, and the neutral findings may be a result of type II error. We continued this study during the COVID-19 pandemic, which required a change in our primary outcome due to an anticipated lack of power to detect important differences in DAOOH as well as several adaptations at our sites. The primary adaptation was to avoid patients with COVID-19 and having to screen out such patients. The other major change was lower overall sample size. Initially, we had hoped to enroll more patients; however, the pandemic had multiple phases that led to multiple interruptions, with differences by site in terms of what was allowed from a research enrollment standpoint. COVID-19 also affected our ability to complete the CONSORT diagram. With so many patients presenting with shortness of breath to the ED, combined with multiple changes in research allowances during the pandemic, we do not have robust numbers to describe the number of patients initially screened. Another limitation was lower-than-expected completion of the KCCQ-12. In some patients for whom the EHR was used to assess vital status, the KCCQ-12 was not completed despite attempts to connect. Such EHR review also leads to another limitation: Patients who died outside of the reach of the EHR may not have been accounted for in our follow-up. Despite these important challenges, our results directionally align with past work showing the safety and potential efficacy of an SSU strategy.^13,14^ We also did not assess resource use and caregiver burden. Although patients may have gone home, whether this benefit is offset by increased resource use or caregiver burden is unknown. Finally, out-of-pocket costs were not explored due to truncated enrollment.

## Conclusions

In this randomized clinical trial, SSU management did not result in significant differences in KCCQ-12 scores but did increase DAOOH at 30 days. Additionally, we did not find any significant differences in adverse events. Lower-than-expected enrollment during the COVID-19 pandemic left the study underpowered to detect a significant difference in KCCQ-12 scores. Our findings suggest that lower health care utilization in the SSU strategy needs to be definitively tested in an adequately powered study. Further randomized clinical trial data are needed, properly powered for these efficacy outcomes, to determine whether there is incremental benefit of SSU treatment vs hospitalization.

## Notes

Supplement 1.
Trial Protocol

Supplement 2. **eTable 1.** Eligibility Criteria **eTable 2.** Study Sites **eTable 3.** Study Outcomes **eTable 4.** KCCQ Power Calculations **eTable 5.** Comparison of Treatment Groups With Both Discharge and 30-Day KCCQ **eTable 6.** ED AHF Medications **eTable 7.** Day of Discharge Medications **eTable 8.** 90-Day Secondary Outcomes **eTable 9.** SSU Failures Compared With Hospitalization Study Group

Supplement 3.
Data Sharing Statement
