# Supplementary material for: Short-Stay Units vs Routine Admission From the Emergency Department in Patients With Acute Heart Failure: The SSU-AHF Randomized Clinical Trial
Source: JAMA Netw Open. 2024 Jan 10;7(1):e2350511. doi: 10.1001/jamanetworkopen.2023.50511 (PMC10782263; doi:10.1001/jamanetworkopen.2023.50511)
Supplement: Supplement 1. — Trial Protocol [file jamanetwopen-e2350511-s001.pdf]

Using Short Stay Units Instead of Routine Admission to Improve Patient Centered Health Outcomes for AHF Patients

Principal Investigator: Peter S. Pang MD

IND/IDE Sponsor: NA

Funded by: AHRQ

Version Number: 6.0

Sept 6, 2017 V3.2 A001

1. Clarification of eligibility criteria as well as adverse events.
2. Aug 16: clarified treatment algorithm
3. 3.0: Clarification of AE's
4. Schedule of events table – Typo corrected

Nov 25, 2017 V3.9 A005

1. Deleted CBQ-HF and added two other Caregiver Burden Scales,
2. New caregiver scales added to schedule of events at baseline and 30 days.
3. Removed the Cr(creatinine lab) requirement from the exclusion criteria and
4. excluded patients hospitalized within 30 days of enrollment.
5. Clarified what data is collected if a patient is hospitalized from the SSU.

Feb 16, 2018 V4.0 A007

1. Clarified eligibility criteria regarding the 30 day exclusion.
2. Clarified that patients on baseline oxygen requirement are still eligible.
3. Endpoint clarification for patients randomized to SSU

Feb 26, 2018 V4.1 A008

1. Modified Bakas Caregiving Outcomes Scale
2. Payment section updated

May 9, 2018 R001 V5.0

1. Changed Systolic Blood Pressure from >115 to >110 in the inclusion criteria
2. Addition to inclusion criteria: "That if vital signs improve, the subject can be considered eligible."
3. Addition to inclusion criteria: "The last heart rate and systolic blood pressure should be within 1 hour of randomization."
4. Removed BUN exclusion criteria
5. Changed Sodium < 135 to Sodium ≤ 131

July 12, 2019 V 6.0

1. Changed Systolic Blood Pressure from >110 to >100 in the inclusion criteria.

2. Removed "Oxygen saturation >93% on room air" from inclusion criteria.

3. Changed GFR <30 to <20 in exclusion criteria

4. Removed "Hemoglobin <9, Sodium ≤ 131, GFR <305. Changed caregiver consenting procedures to include verbal consent if acceptable from individual IRBs.

6. Added repleting potassium to expectedness within Adverse events.

7. Changed caregiver consent to verbal consent from written informed consent. No medical record review of caregiver, purely survey questions.

Table of Contents

LIST OF ABBREVIATIONS 7
STATEMENT OF COMPLIANCE 7
PROTOCOL SUMMARY 8
SCHEMATIC OF STUDY DESIGN 10
1 KEY ROLES 10
2 INTRODUCTION: BACKGROUND INFORMATION AND SCIENTIFIC RATIONALE 10
2.1 Background Information 10
2.2 Rationale 11
2.3 Potential Risks and Benefits 11
2.3.1 Known Potential Risks 11
2.3.2 Known Potential Benefits 11
3 OBJECTIVES AND PURPOSE 12
4 STUDY DESIGN AND ENDPOINTS 12
4.1 Description of the Study Design 12
4.2.1 Primary Endpoint 12
4.2.2 Exploratory Endpoints 12
5 STUDY ENROLLMENT AND WITHDRAWAL 12
5.1 Participant Inclusion Criteria 12
5.2 Participant Exclusion Criteria 12
5.3 Strategies for Recruitment and Retention 13
5.4 Participant Withdrawal or termination 14
5.4.1 Reasons for Withdrawal or Termination 14
5.4.2 Handling of Participant Withdrawals or termination 14
5.5 Premature Termination or Suspension of Study 14
6 STUDY AGENT 14
6.1 Study Agent(s) and Control Description 14
6.1.1 Dosing & Dose escalation 14
6.1.2 Duration of Therapy 16
7 STUDY PROCEDURES AND SCHEDULE 16
7.1 Study Procedures/Evaluations 16
7.1.1 Study specific procedures 16
7.1.2 Standard of care study procedures 16
7.2 Laboratory Procedures/Evaluations 16
7.2.1 Clinical Laboratory Evaluations 16
7.2.2 Other Assays or Procedures .... **Error! Bookmark not defined.**
7.2.3 Specimen Preparation, Handling, and Storage .... **Error! Bookmark not defined.**
7.2.4 Specimen Shipment .... **Error! Bookmark not defined.**
7.3 Study Schedule 16
7.3.1 Screening 16
7.3.2 Enrollment/Baseline 17
7.3.3 Follow-up & Final study visit 17

7.3.7 Schedule of Events Table ... 18
7.5 Concomitant Medications ... 18
7.6 Prohibited Medications, Treatments, and Procedures ... 19
8 ASSESSMENT OF SAFETY ... 19
8.1 Specification of Safety Parameters ... 19
8.1.1 Definition of Adverse Events (AE) ... 19
8.1.2 Definition of Serious Adverse Events (SAE) ... 19
8.2 Classification of an Adverse Event ... 20
8.2.1 Severity of Event ... 20
8.2.2 Relationship to Study Agent ... 20
8.2.3 Expectedness ... 20
8.3 Time Period and Frequency for Event Assessment and Follow-Up ... 21
8.4 Reporting Procedures ... 21
8.4.1 Adverse Event Reporting ... 21
8.4.2 Serious Adverse Event Reporting ... 21
8.5 Study Halting Rules ... 21
8.6 Safety Oversight ... 21
9 CLINICAL MONITORING ... 21
10 STATISTICAL CONSIDERATIONS ... 21
10.1 Statistical and Analytical Plans ... 21
10.2 Statistical Hypotheses ... 21
10.3 Analysis Datasets ... 23
10.4 Description of Statistical Methods ... 24
10.4.1 General Approach ... 24
10.4.2 Analysis of the Primary Efficacy Endpoint(s) ... 24
10.4.3 Analysis of the EXPLORATORY Endpoint(s) ... 24
10.4.4 Safety Analyses ... 29
10.4.5 Adherence and Retention Analyses ... 29
10.4.6 Baseline Descriptive Statistics ... 29
10.4.7 Planned Interim Analyses ... 30
10.5 Sample Size ... 30
10.6 Measures to Minimize Bias ... 30
10.6.1 Enrollment/ Randomization/ Masking Procedures ... 30
11 SOURCE DOCUMENTS AND ACCESS TO SOURCE DATA/DOCUMENTS ... 30
12 QUALITY ASSURANCE AND QUALITY CONTROL ... 31
13 ETHICS/PROTECTION OF HUMAN SUBJECTS ... 32
13.1 Ethical Standard ... 32
13.2 Institutional Review Board ... 32
13.3 Informed Consent Process ... 32
13.3.1 Consent/assent and Other Informational Documents Provided to Participants 32
13.3.2 Consent Procedures and Documentation ... 32
13.4 Participant and data Confidentiality ... 32
14 DATA HANDLING AND RECORD KEEPING ... 32

14.1 Data Collection and Management Responsibilities 33
14.2 Study Records Retention 33
14.3 Protocol Deviations 33
14.4 Publication and Data Sharing Policy 33
15 STUDY ADMINISTRATION 36
15.1 Study Leadership 36
16 CONFLICT OF INTEREST POLICY 37
17 LITERATURE REFERENCES 37

LIST OF ABBREVIATIONS

AHF	Acute Heart Failure
US	United States
HF	Heart Failure
ED	Emergency department
EP	Emergency Physician
DAOOH	Days alive and out of hospital
NIV	Non-invasive ventilation (positive pressure ventilation)
NTG	Nitroglycerin
IV	Intravenous
SL	Sub-lingual
AKI	Acute Kidney Injury
WRF	Worsening renal function
WHF	Worsening heart failure
AE	Adverse event
SAE	Serious Adverse Event
SBP	Systolic Blood Pressure
Hgb	Hemoglobin
Hct	Hematocrit
IEC	Institutional Ethics Committee
IRB	Institutional Review Board
GCP	Good clinical practice
SSU	Short Stay Unit
OU	Observation Unit
QoL	Quality of Life

STATEMENT OF COMPLIANCE

This study will be conducted in full accordance with the Good Clinical Practice: Consolidated Guideline approved by the International Conference on Harmonization (ICH) and any applicable national and local laws and regulations (e.g., Title 21 Code of Federal Regulations [21CFR] Parts 50, 54, 56, 312, and 314). Any episode of noncompliance will be documented.

The Investigators are responsible for performing the study in accordance with this protocol and the ICH and Good Clinical Practice (GCP) guidelines and for collecting, recording, and reporting the data accurately and properly. Agreement of each Investigator to conduct and administer this study in accordance with the protocol will be documented in separate study agreements with the sponsor and other forms as required by national authorities.

Each Investigator is responsible for ensuring the privacy, health, and welfare of the patients during and after the study and must ensure that trained personnel are immediately available in case of a medical emergency.

The Principal Investigator at each center has the overall responsibility for the conduct and administration of the study at that center and for contacts with study management, with the Independent Ethics Committee/Institutional Review Board (IEC/IRB), and with local authorities.

INVESTIGATOR AGREEMENT

I have read this protocol and agree:

- To conduct the study as outlined herein, in accordance with current Good Clinical Practices (GCPs), the guiding principles of the Declaration of Helsinki and complying with the obligations and requirements of Clinical Investigators and all other requirements listed in 21 CFR Part 312, local regulations, and according to the study procedures provided by Indiana University
- Not to implement any changes to the protocol without prior agreement from Indiana University and prior review and written approval from the IRB/EC, except as would be necessary to eliminate an immediate hazard to study patient(s), or for administrative aspects of the study.
- To ensure that all persons assisting me with the study are adequately informed about their study-related duties as described in the protocol.
- To completely inform all patients in this study concerning the pertinent details and purpose of the study prior to their agreement to participate in the study in accordance with GCP and regulatory authority requirements.
- That I will be responsible for maintaining each patient's consent form in the study file and provide each patient with a signed copy of the consent form.

Investigator Name and Title: ____

____

Institution Address: ____

____

____

Signature: ____ Date: ____________

PROTOCOL SUMMARY

Title: Using Short Stay Units Instead of Routine Admission to Improve Patient Centered Health Outcomes for AHF Patients

Précis: Of the over one million annual AHF hospitalizations, approximately 80% are initially treated in the emergency department (ED).[1,2] AHF is the most common and costliest reason why patients over the age of 65 are hospitalized.[3] While some clearly require admission, others do not.[4,5] There is a spectrum of risk for ED patients with AHF. Previous reports suggest 50% of patients could be discharged from the ED or observed for less than 24 hours.[4] Yet admission rates from the ED for AHF remain around 80%.[6] From a patient perspective, we know patients and caregivers would prefer to spend as little time as safely possible in the hospital

Using a multi-center, randomized controlled design, this clinical effectiveness trial will test whether Short Stay Unit AHF management for < 24 hours increases DAOOH and QoL compared to usual care, defined as in-hospital management.

Objectives:

Aim 1*:* To demonstrate the effectiveness of a SSU AHF management strategy vs. usual AHF care (i.e. inpatient admission), using a randomized, controlled, simple, trial design.

Aim 2: To demonstrate that a SSU AHF strategy of care leads to equivalent or improved adherence to HF guidelines at time of discharge vs. usual care (i.e. hospitalization).

Endpoints

Primary Endpoint: Days alive and out of hospital at 30 days post-discharge.

Secondary Endpoints: 1) Quality of life as measured by Kansas City Cardiomyopathy Questionnaire 2) Cost-effectiveness analysis between the two arms at 30 days

Exploratory Endpoints: 1) Caregiver burden as measured by two Caregiver Burden Scales at 30 days; 2) Cost-Effectiveness of the SSU AHF strategy of care at 90 days; 3) Resource utilization measured by the Modified Resource Utilization Questionnaire for Heart Failure (mRUQ-HF) at 90 days 4) All cause mortality and re-hospitalization at 30 and 90 days 5) Days alive and out of hospital at 90 days 6) HF Guideline adherence at time of discharge

**Population:**	ED patients with AHF who meet inclusion and no exclusion criteria will be enrolled once written informed consent is obtained.
**Phase:**	NA
**Number of Sites enrolling participants:**	Four. Projected sample size, n=534.
**Description of Study Agent:**	SSU AHF care for < 24 hours vs. usual care (inpatient admission) in ED patients with AHF who lack baseline high-risk features.
**Study Duration:**	4.5 years. There will be three months of start up, and three months of study conclusion work. Enrollment will occur over 48 months.
**Participant Duration:**	90 days post discharge

SCHEMATIC OF STUDY DESIGN

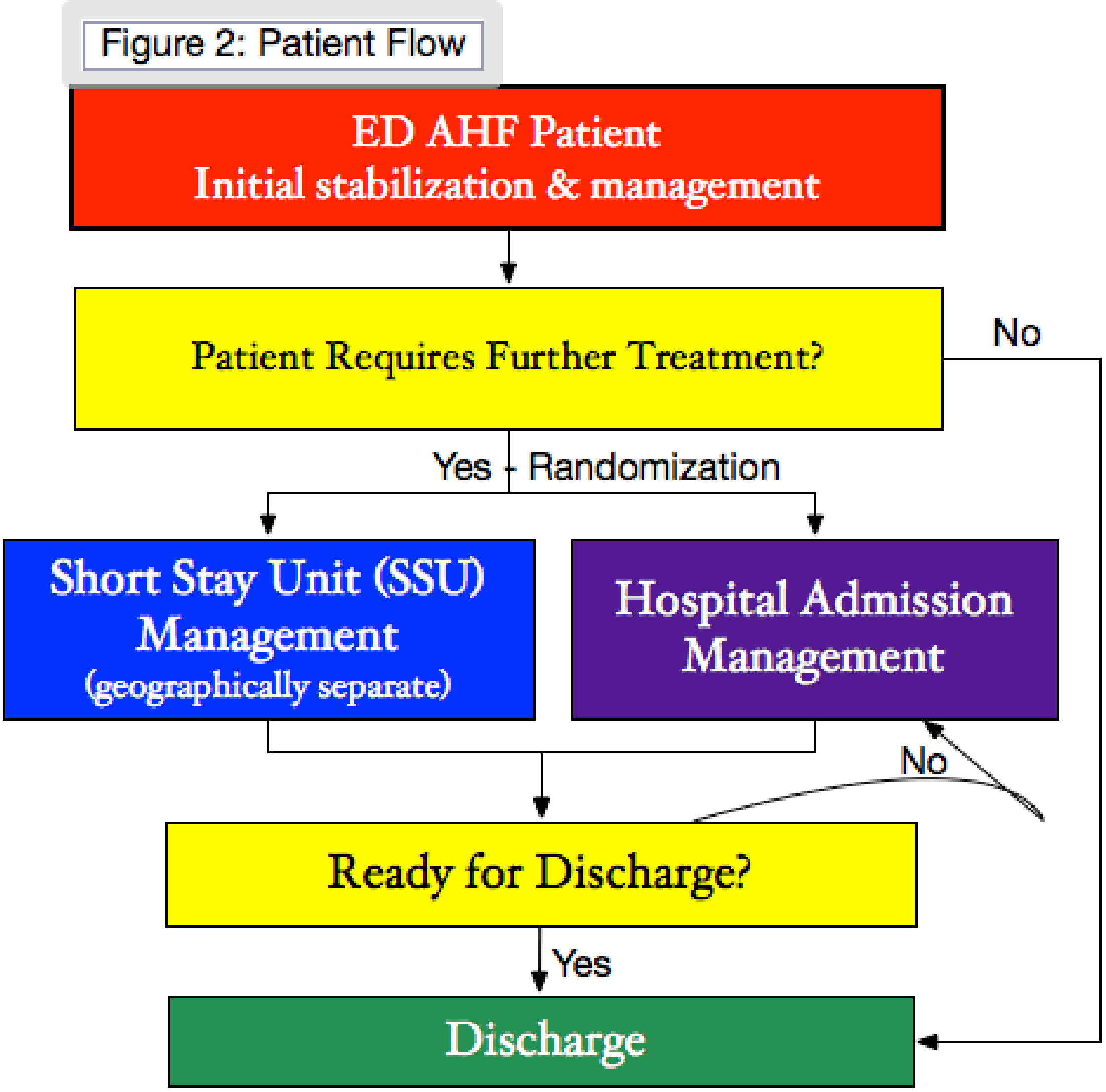

1 KEY ROLES

Our team of investigators is uniquely qualified to successfully complete this study. We leverage complementary experience and expertise, in particular, early (ED) enrollment, short stay unit (SSU) management, and clinical trials. Most importantly, we have worked close together for nearly 10 years.[5,7-29]

Peter S. Pang MD (PI) is an Associate Professor in Emergency Medicine at the Indiana University School of Medicine (IU SOM).

Xiaochun Li PhD, from the Department of Biostatistics at Indiana University School of Medicine will lead the Data Core at IU.

Sean Collins MD (Vanderbilt University), Professor and Vice-Chair of Research, will the site PI at Vanderbilt.

Susan Pressler PhD is a Professor of Nursing at the Indiana University School of Nursing. She helped develop the modified Resource Utilization Questionairre in Heart Failure and is an expert in quality of life assessments.

2 INTRODUCTION: BACKGROUND INFORMATION AND SCIENTIFIC RATIONALE

2.1 BACKGROUND INFORMATION

Heart failure is a major public health burden. Over 5.5 million Americans have heart failure (HF), with 870,000 new diagnoses each year.[30] Within 5 years, half of these patients will be dead.[30] By 2030, the prevalence of HF will increase 46% and HF costs will increase, from $31 billion to $70 billion.[31] Hospitalization for AHF consumes the majority of these dollars.[32,33] HF is the most common reason why patients over the age of 65 are hospitalized.[3] Hospitalization is not benign, marking patients at greatest risk for adverse outcomes.[33,34] Re-hospitalized patients are at even greater risk.[34] After hospitalization, patients enter a *vulnerable phase*.[35-38] During this vulnerable phase, approximately 20% of hospitalized patients experience another medical adverse event.[35] Not only patients are affected; hospitalization/rehospitalization takes a toll upon families and caregivers.[35]

The emergency department (ED) accounts for the majority of the one million hospitalizations for AHF annually. Approximately 80% of AHF admissions are initially treated in the ED.[1,2] Unlike chronic HF, no AHF treatment definitively prolongs life or reduces the risk for re-hospitalization.[39] Clearly some patients require admission, however a large proportion do not require prolonged hospitalization.[4,5] Many AHF patients have congestion secondary to worsening chronic HF. These patients receive IV diuretic therapy and little additional pharmacologic management, despite a median length of stay of 4.3 days.[1,22,25,40-42] This is one reason institutions advocate for outpatient 'infusion centers' or 'diuretic clinics.'[43] Admitting and re-admitting lower risk AHF patients who don't need it may increase their risk for poor outcomes and decrease their quality of life. **Safe alternatives to hospitalization from the ED are needed.** A recent NHLBI working group identified alternatives to hospitalization from the ED as a high priority.[15] The Institute of Medicine identified observation units as an opportunity to improve patient flow resource utilization.[44]

2.2 RATIONALE

Small studies, retrospective analyses, and the Society for Cardiovascular Patient Care (SCPC; now American College of Cardiology [ACC] Accreditaiton Services) guidelines support brief observation (< 24 hours) or short stay unit (SSU) management of AHF to be effective for lower risk AHF patients. Our group's early work demonstrated lower overall median length-of-stay compared to a risk-matched inpatient only group (25.7 vs 58.5 hours).[45] Importantly, total charges were approximately $3600 less for the SSU compared with those admitted from the ED. In a retrospective study of 358 patients using SCPC guidelines, the authors observed less hospital bed day utilization and similar 30-day readmission rates (12.5% vs 10.0%), respectively after adjustment for age, race, sex, BNP, renal function and ejection fraction.[46] SSU discharges spent 2.4 days and 2.5 days within 30 and 90 days of follow-up, respectively, compared with 4.4 and 5.0 days among patients admitted to the hospital after SSU management ($p<0.0001$).[46] Those patients who weren't ready for discharge after SSU management were admitted to the hospital for further treatment. **However, several knowledge gaps hinder broad uptake of SSU management.** First, an adequately powered, randomized controlled trial of SSU AHF care has not yet been conducted. Second, the impact of a SSU AHF strategy on quality of life and caregiver burden is unknown. Third, due to reimbursement differences, patients may face increased out-of-pocket costs. Finally, clinicians and caregivers may think SSU is just 'gaming the system' to avoid readmission penalties. We address each of these gaps in our proposal.

2.3 POTENTIAL RISKS AND BENEFITS

2.3.1 KNOWN POTENTIAL RISKS

Patients will only receive current usual care for acute heart failure. There will be no experimental drugs or therapies. However, the SSU are will receive protocolized care while the usual care arm will receive the non-protocolized AHF care per routine. Past small studies and retrospective analyses demonstrate the safety and efficacy of SSU management. Furthermore, if SSU treated patients are not ready for discharge, they will be admitted. Thus, there is a built in 'safety-net.' However, the SSU strategy has not been tested in a randomized, controlled, clinical trial. Nevertheless, there is a risk that patients will be discharged prematurely from either setting.

2.3.2 KNOWN POTENTIAL BENEFITS

Patients enrolled in this study who are receiving the strategy-of-care or usual care may receive a benefit such as decreased mortality, or less frequent hospitalizations related to HF, or leave the hospital sooner, or improved quality of life. If this study is positive, future patients may benefit from avoiding unnecessary time in the hospital, but equal or better outcomes.

3 OBJECTIVES AND PURPOSE

Objectives:

1: To demonstrate the effectiveness of a SSU AHF management strategy vs. usual AHF care (i.e. inpatient admission), using a randomized, controlled, simple, trial design.

2. To demonstrate that a SSU AHF strategy of care leads to equivalent or improved adherence to HF guidelines at time of discharge vs. usual care (i.e. hospitalization).

Purpose:

The overarching goal of this comparative effectiveness study is to increase days-alive-and-out-of-the-hospital (DAOOH) and improve quality of life (QoL) for lower risk AHF patients who present to the emergency department (ED). We hypothesize an alternative to hospitalization, SSU AHF management of less than 24 hours, will achieve our goal.

4 STUDY DESIGN AND ENDPOINTS

4.1 DESCRIPTION OF THE STUDY DESIGN

A prospective, randomized, controlled, simple, comparative effectiveness study of a strategy of care (SSU care) vs. usual care (inpatient admission) in ED patients with AHF who lack baseline high-risk features and ineligible for ED discharge.

4.2.1 PRIMARY ENDPOINT

Days alive and out of hospital at 30 days post-discharge.

4.2.2 SECONDARY ENDPOINTS

1) Quality of life as measured by Kansas City Cardiomyopathy Questionnaire 2) Cost effectiveness analysis between the two arms at 30 days.

4.2.3 EXPLORATORY ENDPOINTS

1) Caregiver burden as measured by **Oberst Caregiving Burden Scale and Bakas Caregiving Outcomes Scale** ; 2) Cost-Effectiveness of the SSU AHF strategy of care at 90 days; 3) Resource utilization measured by the Modified Resource Utilization Questionnaire for Heart Failure (mRUQ-HF) 4) All cause mortality at 30 and 90 days 5) All cause re-hospitalization at 30 and 90 days; 6) Days alive and out of hospital at 90 days 7) HF Guideline adherence at 30 days

5 STUDY ENROLLMENT AND WITHDRAWAL

5.1 PARTICIPANT INCLUSION CRITERIA

1) ED physician clinical diagnosis of AHF;
2) Planned admission for AHF
3) Systolic blood pressure > 100mmHg*, heart rate < 115bpm. The last HR and SBP should be within 1 hour of randomization.
4) Previous history of HF

*Patients with atrial fibrillation but controlled HR are eligible

For Caregiver Burden assessments. The eligibility criteria for a caregiver: 1) person either self-identifies, or when asked identifies themselves, as the primary caregiver for the patient. If there are multiple caregivers, the person who self-identifies as providing the most care will be asked to provide verbal informed consent. This part of the investigation pses no more than minimal risk to the subject and caregivers will be provided with information about the study and the processes for the initial and 30 day follow up questionnaires. For patients with no caregiveridentified, no caregiver assessment will be performed.

5.2 PARTICIPANT EXCLUSION CRITERIA

1) Patients hospitalized within the last 30 days ONLY if the institution mandates these patients are observed. Otherwise, these patients remain eligible.
2) Transplanted organ of any kind or ventricular assist device patient;
3) End stage renal disease, on dialysis, or eGFR < 20 mL/min;
4) Acute coronary syndrome (e.g. EKG changes consistent with ischemia or troponin elevation secondary to ACS as per the treating ED clinician);
5) Other acute co-morbid conditions (e.g. sepsis, altered mental status);

6)Patients who require ventilatory support of any kind or intravenous vasodilators/vasopressor/inotropic support at the time of ED disposition

7) Pregnant patients or any patient who has been pregnant in the last 3 months

8)≤ 18 years of age

9)Any patient who in the opinion of the clinician or investigator should not be in an obs unit or requires ICU level care or will require inpatient rehabilitation or skilled nursing facility after discharge from the ED or hospital

10) Planned discharge from the emergency department

11) De novo (new onset) AHF

5.3 STRATEGIES FOR RECRUITMENT AND RETENTION

Sites will be initiated only after IRB approval and site training. Each site has well-established procedures for identifying potential subjects for AHF clinical trials. Broadly, these are based on electronic screening of ‘tracking boards’ in the ED, alert systems generated by the electronic health record, and direct interaction with ED caregivers. Each site will also be required to utilize a *‘boots-on-the-ground’ approach. While electronic aids may be used, each site must have research staff physically walk through the ED frequently throughout the day or stationed in the ED.* Patients with AHF will be pre-screened by dedicated, trained, experienced research personnel to determine if eligibility criteria are met. Patients will then be approached to ascertain their willingness to participate after discussion with the patient’s primary caregiving team. Only after written informed consent will randomization occur.

As a comparative effectiveness trial, the inclusion/exclusion criteria are relatively broad compared to other therapeutic clinical trials.

Screen Failures:

Patients who sign an informed consent but who are not randomized, or after randomization have a change in clinical status making them ineligible, will be considered Screen Failures. Only data for randomized patients will be entered into the CRF. Serious adverse events should be reported for these patients from the time the ICF is signed through the time

that the patient is declared a screen failure. One expected reason for screen failure will be a change in plan to discharge the patient from the ED.

Minimizing loss to follow-up (LTFU), as well as completion of 30-day and 90-day vital status assessments and QoL/Caregiver Burden questionnaires, are critical to study success. Every effort will be made to ensure follow-up with patients and caregivers at 30 and 90 days, including phone call, text messaging, email, regular mail, and electronic health record review. Based on the extensive experience at each site, we do not anticipate a large LTFU rate. However, our sample size calculations account for attrition (see below).

5.4 PARTICIPANT WITHDRAWAL OR TERMINATION

5.4.1 REASONS FOR WITHDRAWAL OR TERMINATION

In accordance with the guiding principles of the Declaration of Helsinki, any patient is free to withdraw from participating in this study at any time and for whatever reason, specified or unspecified, and without prejudice. Investigators should attempt to determine the cause of withdrawal and, if desired by the patient, to make it possible for the patient to continue to participate in the study. The extent of a patient's withdrawal from the study (i.e. withdrawal from further study treatment, withdrawal from any further contact, etc.) should be documented. Every effort should be taken to follow all randomized patients, to the extent that the patient will allow, for the full follow-up period.

Investigators may discontinue study treatment for any other reasons concerning the health or well-being of the patient.

The reason for and date of study discontinuation and the reason for and date of withdrawal from the study must be recorded on the CRF. If study is discontinued because of an adverse event or a clinically significant abnormal laboratory test result, evaluations will continue until the event has resolved or stabilized or until a determination of a cause unrelated to the study procedure is made. The specific event or laboratory finding(s) must be documented. All evaluations should be performed, according to the protocol.

5.4.2 HANDLING OF PARTICIPANT WITHDRAWALS OR TERMINATION

The Full Analysis Set (FAS) will include all randomized patients. In accordance with the intent-to-treat principle, patients will be analyzed by the group to which they were randomized. Misrandomized patients (patients randomized in error who did not receive any study intervention) will be excluded. Analyses in the FAS will constitute the main efficacy results for the primary and secondary study efficacy endpoints.

The Per Protocol Set (PPS) will be a subset of the FAS and will exclude patients with major protocol violations.   The major protocol violations that will result in exclusion from the PPS will be identified. Patients will be analyzed in the treatment group to which they were randomized. Results of analyses in this analysis population will support the primary efficacy analyses in the FAS.

5.5 PREMATURE TERMINATION OR SUSPENSION OF STUDY

The study is overseen by a DSMB. They may terminate the study at any time if the safety of patients is at jeopardy.

6 STUDY AGENT

6.1 STUDY AGENT(S) AND CONTROL DESCRIPTION

This study will test a strategy-of-care, SSU AHF management for less than 24 hours vs. usual care (hospitalization) for lower risk AHF patients who are planned to be admitted. Only therapies commonly utilized during usual AHF care will be applied. For example, , IV loop diuretics, and topical and oral vasodilators. Usual care will be at the discretion of the clinical care team. No drugs or therapies that are not approved by the FDA will be allowed.

6.1.1 TREATMENT PROTOCOL

The algorithm below outlines the SSU AHF protocol.

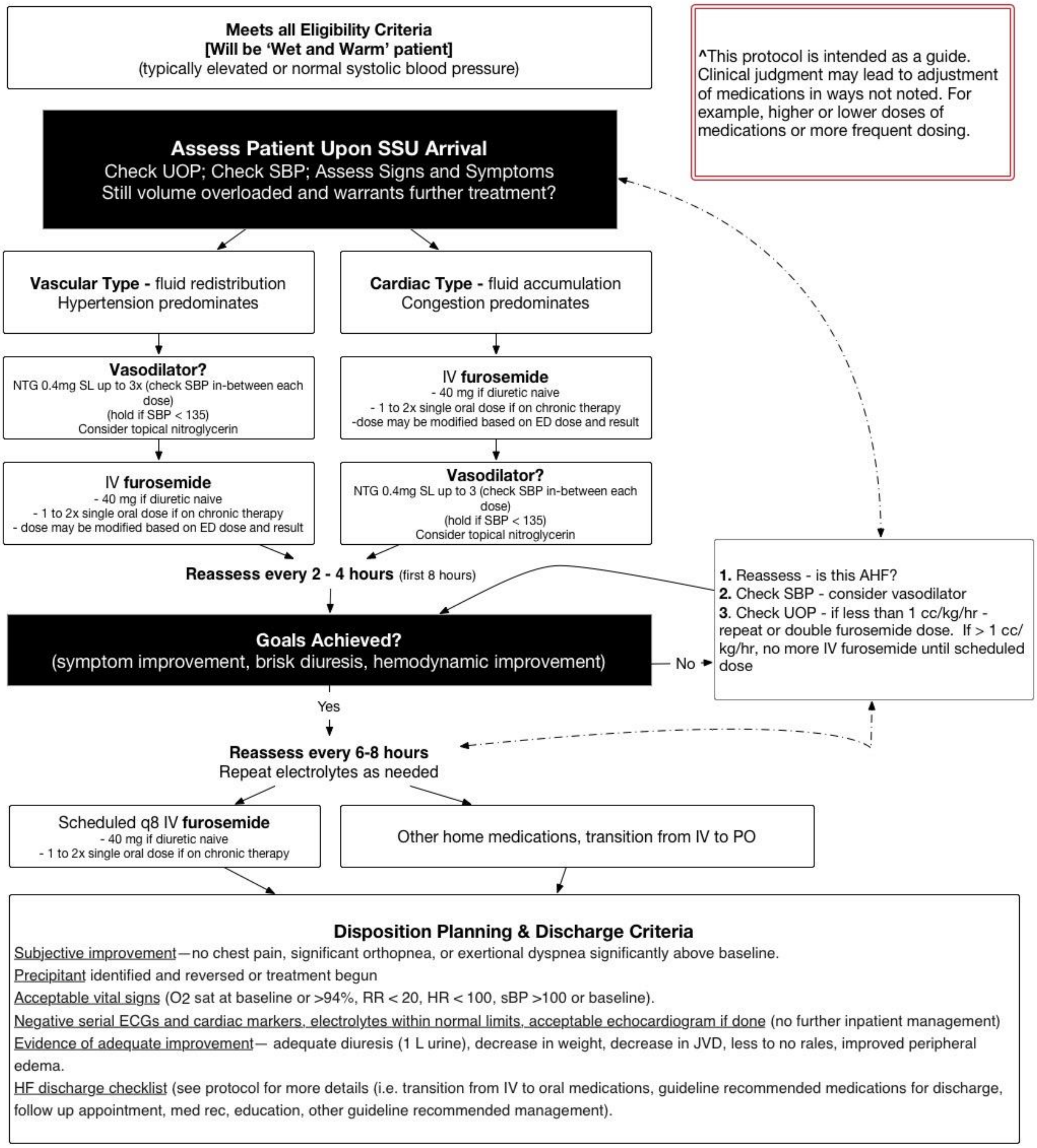

Arm 1. SSU AHF Strategy: 'Short stay' is defined as < 24 hours. All SSU are monitored beds. Nursing ratios are similar to telemetry wards. Four principles of SSU AHF management are outlined: 1) relief of HF symptoms and signs; 2) decongestion while observing and correcting any electrolyte imbalances; 3) hemodynamic improvement; and 4) robust care transitions with an emphasis on guideline directed medication reconciliation and guideline recommended therapy at discharge.[16,46] One potential advantage of the SSU AHF strategy is protocol driven care. (See Figure 3, next page) This figure is adapted from the ESC and SCPC Guidelines, primarily adding greater detail regarding vasodilator and diuretic dosing.[47] "Warm and wet" defines the HF phenotype from the Nohria et.al. classification: well perfused, volume overloaded.[48] The discharge criteria are taken from the SCPC guidelines on observation unit care.[16]

Arm 2. Usual care (defined by inpatient admission for AHF) was chosen as the comparator. In 2009, Dawson et.al. published the background framework for the 2005 NIH funded meeting on "Considering Usual Care in Clinical Trial Design: Scientific and Ethical Issues."[49] These principles informed our decision to use 'usual care' as the comparator.

One specific principle: consideration of 'usual care' when a comparator hypothesizes to be better than or equivalent to current clinical practice.[49] Hospitalization is the dominant strategy of care for over 80% of patients who present to the ED with AHF; thus, it provides the best real-world comparison of AHF care.[50] The median length of stay is 4.3 days.[1] We expect nearly all usual care patients to be cared for on general wards or telemetry and not ICU level of care, given our lower risk eligibility criteria. Guidelines are clear on suggested best practices for management.[49] Admittedly, the evidence supporting these statements in AHF are not as robust as chronic HF. This is one reason usual care has frequently been used in AHF therapeutic clinical trials.[51-53] Nevertheless, we will encourage guideline adherence in BOTH arms of the trial, both during hospitalization and pre-discharge. As hospitals are penalized on excessive 30-day readmissions, each participating hospital has a transitional care plan. In keeping with the pragmatic study design, each study arm will utilize their existing transitional care programs.

6.1.2 DURATION OF THERAPY

Both arms will continue until patients are deemed ready for discharge, either from the SSU or the hospital. For patients deemed not ready for discharge after the SSU management phase, will be admitted.

7 STUDY PROCEDURES AND SCHEDULE

7.1 STUDY PROCEDURES/EVALUATIONS

7.1.1 STUDY SPECIFIC PROCEDURES

The table below in section 7.3.7 highlights study specific procedures. Only patients who sign written informed consent will under study specific procedures.

7.1.2 STANDARD OF CARE STUDY PROCEDURES

There will be no other change to standard of care procedures for either treatment arm. Patient care and safety are paramount and trump all other considerations.

7.2 LABORATORY PROCEDURES/EVALUATIONS

7.2.1 CLINICAL LABORATORY EVALUATIONS

Lab testing will be based on standard practice and likely include analyzed by the clinical lab at each respective institution for baseline chemistry and hemoglobin/hematocrit values. This reflects our pragmatic approach. Only labs collected as part of usual or standard care will be collected. No lab testing will be done solely for research purposes.

If drawn, all clinical laboratory test results outside of the reference range will be interpreted in the context of the patient underlying disease state by the Investigator using the following categories:

- abnormal but not a clinically-significant worsening
- abnormal and a clinically-significant worsening

A local laboratory will be utilized to analyze screening entry criteria. Laboratory tests not specified in the protocol but required by the Investigator to assess patient care will be performed at the local laboratory in accordance with Standard of Care.

7.3 STUDY SCHEDULE

7.3.1 SCREENING

A signed and dated informed consent form will be obtained before any study-specific screening procedures are performed. Results of evaluations obtained as part of routine medical care, which are performed prior to obtaining

informed consent, may be used in place of the protocol-specified evaluations. Patients will acknowledge and agree to the use of this information for the study by giving informed consent.

At the Baseline Visit, patients will be assigned by the IWRS a unique permanent identification number (referred to as the patient identification number) such that all randomized patients from each center are given consecutive identification numbers by the IWRS in successive order of inclusion. We will utilize the REDCap randomization module.

Prospective study patients will have presented to the hospital for urgent therapy for AHF. Potential patients will be identified either en route to or upon arriving at the ED/hospital. Routine assessments associated with usual patient care may be used for the purposes of screening and may be completed in any order. Study specific procedures must be completed only after Informed Consent is obtained.

Randomization will occur during the patients ED stay. This may occur at any time during their ED stay.

The following procedures will be performed prior to or during Screening:

- Obtain written informed consent (must be performed as the first study-specific procedure)
- Review and record medical history
- Record prior and concomitant medications
- Physical examination (including height and weight when reasonably possible)
- Vital signs measurements (includes systolic and diastolic blood pressures, heart rate, body temperature, oxygen saturation reading and respiratory rate)
- 12-Lead Electrocardiogram per standard practice
- Blood collection for local laboratory tests, including BNP or NT-proBNP, and pregnancy test if applicable per local standard practice.
- Inquiry about Adverse events

7.3.2 ENROLLMENT/BASELINE

Patients who continue to fulfill all of the inclusion/exclusion criteria will be randomized. Randomization will occur via central IWRS system.

7.3.3 FOLLOW-UP & FINAL STUDY VISIT

Patients will have follow up at 30 (+/- 7 days) days and 90 (+/- 30) days post-discharge (up to 120 total days after randomization is allowed if difficult to get a hold of a patient or more time is required to complete forms). There will be NO further in-person visits once discharged. However, treatment during hospitalization will be recorded. Patients will be called 30 days post-discharge ((+) 7 business days) and 90 days post-discharge (+/- 30 days) to assess vital status, re-hospitalizations or ED visits, as well as quality of life, caregiver burden, and resource utilization.

7.3.7 SCHEDULE OF EVENTS TABLE

Schedule of Events Timepoint/Visit	Screen	Baseline	ARM 1 - PreDischarge from SSU	ARM 2 - PreDischarge from Hospital Floor	30-Day & 90 Day Follow up
**Informed Consent**	X				
**Medical History**		X			
**Physical Exam**		X	X	X	
**Clinical lab tests***		X	X~	X~	
**ECG***		X			
**CXR***		X			
**KCCQ (QoL) & SF-12**			X	X	X
**Oberst Caregiving Burden Scale and Bakas Caregiving Outcomes Scale**			X	X	X[1]
**HF Guideline Assessment**			X	X	
**Collect concomitant meds**		X	X	X	
**mRUQ-HF (resource utilization)**					X^
**Cost Effectiveness Measures (i.e. DRG)**					X
**Guideline adherence assessment**		X	X	X	X[1]
**SCPC Discharge Criteria Adherence**			X	X	
**Assessment of AE/SAE's through 5 days**			X	X	
**Vital Status, ED visits, Hospital days, Re-admission status**			X	X	X
**Subject Payment**			X	X	X[2]
*per standard of care. Typical labs include: Na, K, renal function, HgB, troponin, Natriuretic Peptide levels. ~only if performed per usual care					

SOC = standard of care, ED = Emergency Department, QoL = quality of life, HF = heart failure, mRUQ = modified resource utilization questionnaire, EMR = electronic medical record, DRG = diagnosis related group

[1]Only through 30 days

[2]Sites may choose to make a single payment or to divide into 2 payments.

^only at 90 days

NOTE: If patients are hospitalized from the SSU, ONLY the following assessments should be collected. Otherwise, the pre-discharge assessments will apply.

- Physical exam
- Clinical lab tests
- Con meds
- SCPC discharge criteria. This would be conducted twice. Once in SCPS prior to hospitalization and again at discharge.

7.5 CONCOMITANT MEDICATIONS

All medications administered within 14 days prior to and during screening will be recorded in the case report form. Medications that are not specifically prohibited are permitted at the Investigator's discretion.

7.6 PROHIBITED MEDICATIONS, TREATMENTS, AND PROCEDURES

No medications, treatments, or procedures are prohibited unless specifically mentioned in the eligibility criteria. Patient safety and well-being are paramount: Any treatment deemed necessary may be utilized at the investigators discretion should there be any concern for the patients health.

8 ASSESSMENT OF SAFETY

In addition to standard safety monitoring by the sponsor, an independent DSMB will oversee patient safety in the trial. The DSMB will meet as specified in its charter.

8.1 SPECIFICATION OF SAFETY PARAMETERS

Mortality, re-hospitalization, and ED visits through 30 and 90 days will be assessed for safety as well as efficacy

8.1.1 DEFINITION OF ADVERSE EVENTS (AE)

The Investigator and study staff are responsible for detecting and recording AEs and SAEs during scheduled safety evaluations and whenever such information is brought to their attention. This section of the protocol provides definitions and detailed procedures to be followed. During each visit, the Investigator will question the patient about adverse events using an open question, taking care not to influence the patient's answers, e.g. "Have you had any unusual symptoms or medical problems since the last visit? If yes, please describe."

An AE is any unfavorable and unintended sign, symptom, or disease temporally associated with the use of an investigational (medicinal) product or other protocol-imposed intervention, regardless of attribution. It is expected that many HF patients will have events that are commonplace due to their HF and associated comorbidities. Our study plans to focus on AE that are outside of what is typically encountered during an AHF event and the subsequent 90 days.

This includes the following:

• AEs not previously observed in the subject that emerge during the protocol-specified AE reporting period (5 days for anything other than mortality, re-hospitalization, and ED visits)

• Complications that occur as a result of protocol-mandated interventions such as a medication adjustment

• Preexisting medical conditions (other than the condition being studied) judged by the investigator to have worsened in severity or frequency or changed in character during the protocol-specified AE reporting period that are outside of what would be expected for a patient with AHF or HF in the outpatient setting.

For example, a patient upgraded to ICU status from the observation or SSU. Hospitalization, by itself, does NOT count as it is expected to occur for a proportion of SSU patients.

• Abnormal laboratory values that fall into an abnormal range based upon the hospital's laboratory standards, the abnormality was not preexisting prior to enrollment, and the abnormality leads to a new treatment within the AE time frame, with the exception of repleting potassium.

8.1.2 DEFINITION OF SERIOUS ADVERSE EVENTS (SAE)

An AE will be classified as an SAE if:

• It results in death (i.e., the AE actually causes or leads to death).

• It is life threatening (i.e., the AE, in the view of the investigator, places the subject at immediate risk of death. It does not include an AE that, had it occurred in a more severe form, might have caused death).

• It requires or prolongs inpatient hospitalization. Admission from the SSU itself does not count as an SAE, but an expected outcome. However, if in the investigators judgment, admission from the SSU was due to some other complication of the study protocol, rather than an expected outcome, it should be reported as an SAE

SPECIFIC REPORTABLE SAE for SSU/OU arm patients:

- Cardiac arrest

- Need for intubation or positive pressure ventilation

• It results in persistent or significant disability/incapacity (i.e., the AE results in substantial disruption of the subject's ability to conduct normal life functions).

• It is considered a significant medical event by the investigator based on medical judgment (e.g., may jeopardize the subject or may require medical/surgical intervention to prevent one of the outcomes listed above).

8.2 CLASSIFICATION OF AN ADVERSE EVENT

8.2.1 SEVERITY OF EVENT

The severity of each adverse event must be recorded as 1 of the choices on the following scale:

**Mild**	No limitation of usual activities
**Moderate**	Some limitation of usual activities
**Severe**	Inability to carry out usual activities

An AE that is assessed as severe should not be confused with a SAE.

8.2.2 RELATIONSHIP TO STUDY PROTOCOL

Each reported AE will be described by its duration (i.e., start and end dates), regulatory seriousness criteria if applicable, and suspected relationship to study protocol in accordance with definitions set forth at each IRB. In general, these relationships are categorized as likely, possible, unlikely and not related. Experience teaches that gray zone instances will arise, and the site coordinators and PIs will be trained to adjudicate possible SAEs in a systematic fashion. To ensure consistency of SAE causality assessments, investigators will apply the following general guideline:

Yes - There is a plausible temporal relationship between the onset of the AE and administration of the study protocol and the AE cannot be readily explained by the subject's clinical state, inter-current illness, or concomitant therapies; and/or the AE follows a known pattern of response to study drug or the AE abates or resolves upon discontinuation of study drug;

No - Evidence exists that the AE has an etiology other than the study protocol (e.g., preexisting medical condition, underlying disease, inter-current illness, or concomitant medication); and/or the AE has no plausible temporal relationship to the study drug.

Adjudication of each AE will proceed as follows: First, the coordinator will consult the site PI to review the chart. Next, the PI will contact members of the clinical care team to clarify uncertainty related to inadequate documentation. Third, if the PI is unable to decide for certain if an AE or SAE occurred, he or she will have the option of sending a personal health identifier-stripped, written narrative of the event to the other site PIs who will vote up or down as to whether the event constituted an AE or SAE.

8.2.3 EXPECTEDNESS

The following signs, symptoms, observations and events are frequently observed in association with acute heart failure and its treatments are exempted from regulatory reporting (including, but not limited to local IRB) unless known to be caused by, or plausibly caused by spironolactone: dyspnea, orthopnea, paroxysmal nocturnal dyspnea, chest pain, fever, hypoxemia, rapid pulse, rapid respiratory rate, dizziness, syncope, altered mental status, confusion, anxiety, generalized weakness, anorexia, nausea, abdominal pain, back pain, early satiety, vomiting, pneumonia, acute renal failure, repleting potassium, skin infection, cancer, surgery not related to treatment of pulmonary embolism, electrocardiography abnormalities (atrial arrhythmias, ventricular dysrhythmias, right bundle branch block, and ST and T wave changes), elevated troponin level, elevated BNP or NT ProBNP level, high white blood cell count, pulmonary infiltrate, pleural effusion, cardiomegaly, electrolyte imbalances, need for oxygen therapy, need for vasopressor support, need for blood product transfusion, need for mechanical ventilation (invasive or non-invasive), need for physical or occupational therapy, need for analgesia, need for skilled nursing facility upon discharge, need for early follow up with physician, escalation of heart failure therapy, need for cardiac catheterization or PA line placement, need for sleep study.

8.3 TIME PERIOD AND FREQUENCY FOR EVENT ASSESSMENT AND FOLLOW-UP

All AEs and SAEs will be followed through resolution, stabilization, or until the subject is lost-to-follow-up.

The onset and end dates, duration, action taken regarding study drug, treatment administered, and outcome for each adverse event must be recorded on the CRF for randomized patients. The relationship of each adverse event to study procedures, and the severity and seriousness of each adverse event, as judged by the Investigator, must be recorded as described below.

8.4 REPORTING PROCEDURES

8.4.1 ADVERSE EVENT REPORTING

The study period during which AEs must be reported begins after informed consent is obtained and initiation of study treatment and for 5 days after randomization. Patients will be followed out to 90 days for death, ED utilization, and re-hospitlization as part fo the study outcomes, which will also count as safety measures. Subject's hospital discharge summaries will be examined at hospital discharge and all non-exempt AEs will be investigated by examining necessary medical records.

8.4.2 SERIOUS ADVERSE EVENT REPORTING

All SAE's will be reviewed within 48 hours and all AE's within 7 days of discovery. SAE's will be followed through the entire follow up period (i.e. through day 30). Any SAE discovery will be reported to the DCC who will then report to the DSMB. If placebo treated patient, standard reporting to the IRB will occur. If active treated patient, and deemed to be related to drug, the SAE will be reported to the DSMB chair within 7 business days by email, fax, or phone of any fatal or life-threatening adverse event that is unexpected.

<u>15 Calendar Day Written Report</u>

The Investigator will also be required to notify the IRBs and all participating investigators, in a written Safety Report, of any serious, unexpected AE that is considered reasonably or possibly related to the strategy-of-care arm

<u>72 hour reporting</u>

For the discovery of an unexpected serious adverse event thought to be related to study procedures the Investigator(s) will notify the Chair of the DSMB by email within 72 hours.

8.5 STUDY HALTING RULES

Please see separate DSMB Charter

8.6 SAFETY OVERSIGHT

Please see separate DSMB Charter

9 CLINICAL MONITORING

Sites will be remotely monitored. Should the need arise for further investigation, an independent monitor will be appointed to visit sites. Each site has extensive clinical trial experience and the expectation for this need is low. Nevertheless, the PI will visit each site at least once per year for meeting with study staff and random surveillance.

10 STATISTICAL CONSIDERATIONS

10.1 STATISTICAL AND ANALYTICAL PLANS

Data Coordinating Center (DCC): The DCC will be housed at the Indiana University School of Medicine in the Department of Biostatistics, led by Xiaochun Li PhD. She will oversee data management and analysis related activities. The DCC will perform the following activities and functions:

- Overall responsibility for the biostatistics related to the trial. In this function, guidance and leadership will be provided regarding clinical trial methodology as well.

- Facilitate good study communication will be facilitated regarding trial progress and enrollment.
- Trial analysis and trial reports will be generated, including reports on the individual components of the study intervention – at both the site and overall study level – to ensure fidelity of each aspect of the study
- Database reports to monitor accrual as well as data quality, with queries generated to sites as needed
- Liaison with the DSMB and provide biostatistical support.
- Facilitate dissemination of the trial results through analysis and participation in the writing of study reports as well as manuscripts and presentations.
- Develop a Data Management Plan (DMP) in collaboration with participating sites of the study.
- Creation and maintenance of the eCRF
- Monitor patient enrollment and data collection by participating sites.
- Establish database lock procedures and clear change-control procedures for unlocking the database
- Produce protocol violation and deviation reports, missing data reports and data discrepancies reports will be generated. Automated edit checks both within and across CRFs and visit dates will be constructed by the DCC Data Manager. Queries generated will be forwarded by the DCC Data Manager to all participating sites' research coordinators for resolution.

10.1.1 ENDPOINTS AND RATIONALE

To ensure patient-centered endpoints, we twice met with patients and caregivers through two community engagement studios.[54] Patients prefer to be healthy and have a good QoL. If a safe alternative is available, patients prefer not to be hospitalized.

The **primary outcome is days alive and out of hospital (DAOOH) at 30 days post-randomization.** Unlike time-to-event analyses that ignores repeat events and gives equal weight to components of a composite endpoint,[55] DAOOH accounts for both frequency and duration of hospitalization and also weights mortality most heavily.[55,56] This endpoint has also been suggested as a quality metric rather than re-hospitalization alone.[57]

The two secondary outcomes are 1) QoL using the KCCQ. Patients desire not only to live longer, but to live better; or at minimum, not be worse after an acute health care related event.[58] Patients with advanced HF would trade survival time (or days alive) for a better QoL.[58] Our pilot work supports the feasibility of assessing QoL in the acute setting and suggests changes in scores are related to hospital re-visits.[59] We hypothesize that fewer days in the hospital in our SSU arm will also lead to a greater QoL. Interviews of hospitalized AHF patients suggest **they would prefer a shorter hospital stay or avoid an admission altogether,** assuming safety and outcomes are similar or better than hospitalization. Thus, assessing QoL is a key aim.

The KCCQ is a patient-reported, validated survey instrument designed to measure QoL in chronic HF patients.[60] Lower scores equal lower patient-reported QoL. KCCQ scores predict adverse outcomes – namely hospitalization and mortality. Importantly, the KCCQ demonstrates responsiveness to clinical change.[60-62]

A change of five points has been previously shown to be clinically significant, and will be assessed prior to discharge and day 30 post-randomization.[62]

2) Cost effectiveness analysis, described in detail below.

NOTE: For patients randomized to SSU who worsen PRIOR to going to SSU, this will be counted the same as a hospitalization from the SSU.

ADDITIONAL ENDPOINTS

Adherence to guidelines saves lives and reduces hospitalizations and re-hospitalizations. We expect both the primary and secondary outcome will be affected by adherence to guidelines. Advances in medical therapy for HF patients with reduced ejection fraction (EF) prolongs life and decreases hospitalization.[39] Yet a substantial proportion of patients are

either not taking guideline-recommended medications or are on sub-optimal doses.[63,64] We will emphasize and measure guideline adherence for eligible patients at the point of discharge from both study arms as well as during follow up.

Guideline adherence: the following HF measures will be assessed in **eligible** patients:[47,65-67] (1) assessment of left ventricular function within a 12 month period, (2) use of angiotensin-converting enzyme inhibitor or angiotensin receptor blocker or ARNI (angiotensin receptor blocker/neprilysin inhibitor) for left ventricular systolic dysfunction (defined as an ejection fraction < 40%), (3) Use of guideline recommended Beta-blocker for left ventricular systolic dysfunction, (4) use of mineralocorticoid receptor antagonists (5) use of ivabradine (6) use of hydralazine/isosorbide dinitrate (7) provision of discharge instructions, (8) counseling for smoking cessation, and (9) follow up appointment provided within 7 days of discharge. A composite heart failure measure will be created by averaging all quality measures available per patient. Specifically, the *composite score* is defined as the total number of interventions performed divided by the total number of interventions for which the patient was eligible. In addition, we will assess for therapies prescribed inappropriately such as glitazones or ARNI prescribed simultaneously with an ARB.

Decreasing hospital stays may increase caregiver burden. Thus, we will also assess caregiver burden, using both the Oberst Caregiver Burden and Bakas Caregiver Outcome Scales, both validated instruments prior to discharge and again at 30 days.[68] Other measures of caregiver burden in HFhave either not involved input from actual caregivers or missed important conceptual domains.[68] Effective caregivers has been associated with improved quality of life, lower hospitalization rates, and reduced mortality.[68,69] As our primary focus is on the patient, the eligibility criteria for the caregiver will be broad, essentially including any person who after separate written informed consent, acknowledges themselves as the primary caregiver for the patient.

Out-of-pocket costs directly impact patients. As such, we will assess healthcare utilization from a societal perspective using the Modified Resource Utilization Questionnaire for Heart Failure (mRUQ-HF).[70] The mRUQ-HF is a14-item self-report questionnaire of comprehensive lists of choice related to healthcare utilization. (See Appendix trial protocol) Exploratory analysis will occur in two steps: 1) Determination of resources consumed or related; 2) Assignment of 'value' to the resources consumed.[71] This two-step process allows for transparency of assigned costs. Hospital costs will be assigned per individual hospital cost-accounting. Where monetary values are not directly self-reported, Medicare fee schedules will be used for uniformity.

10.2 STATISTICAL HYPOTHESES

Aim 1*:* To demonstrate the effectiveness of a SSU AHF management strategy vs. usual AHF care (i.e. inpatient admission), using a randomized, controlled, simple trial design.

- ***Hypothesis 1a:*** SSU treated patients will have one more DAOOH within 30 days post-randomization vs. usual care arm. Assuming a 10% attrition rate, we will randomize 534 patients 1:1, which will provide 80% power (alpha 0.05, two-sided) to demonstrate a one-day difference in the primary outcome at 30 days post-randomization.
- ***Hypothesis 1b:*** An AHF SSU strategy of care will lead to significant improvement in QoL scores at 30 days post-discharge compared with usual care. QoL will be assessed with the KCCQ.[59]

Aim 2: To demonstrate if a SSU AHF strategy of care leads to equivalent or improved adherence to HF guidelines at time of discharge compared to usual care.

- ***Hypothesis 2:*** Adherence to HF guidelines at time of discharge in the SSU arm is equivalent or better than adherence in the usual care arm.

10.3 ANALYSIS DATASETS

The Full Analysis Set (FAS) will include all randomized patients. In accordance with the intent-to-treat principle, patients will be analyzed by the group to which they were randomized. Misrandomized patients (patients randomized in error

who did not receive any study intervention) will be excluded. Analyses in the FAS will constitute the main efficacy results for the primary and secondary study efficacy endpoints.

The Per Protocol Set (PPS) will be a subset of the FAS and will exclude patients with major protocol violations. The major protocol violations that will result in exclusion from the PPS will be identified. Patients will be analyzed in the treatment group to which they were randomized. Results of analyses in this analysis population will support the primary efficacy analyses in the FAS.

10.4 DESCRIPTION OF STATISTICAL METHODS

10.4.1 GENERAL APPROACH

Unless stated otherwise, two-sided p values < 0.05 will be considered statistically significant, without regard to multiple comparisons. Statistical tables and listings and analyses will be produced using SAS® release 9.1 or later (SAS Institute, Inc, Cary, NC, USA) or other validated statistical software.

10.4.2 ANALYSIS OF THE PRIMARY EFFICACY ENDPOINT(S)

Patient baseline characteristics will be tabulated for both SSU and usual care groups. Specifically, means (standard deviations) or medians (interquartile range) will be presented for continuous variables such as age and body mass index. For categorical variables, such as gender and race, frequency and percentages will be presented. Group comparisons of continuous variables will be based on either two-sample t-tests (under normality) or Wilcoxon's Rank Sum test. Normality of distribution will be determined using the Kolmogorov-Smirnov goodness-of-fit test. Categorical data comparisons will be presented based on the Chi-square or Fisher's exact test. Standardized difference is used to compare the mean of the SSU and usual care groups.[72]

In-hospital and SSU measurements: Data points collected during hospitalization and the SSU (e.g. medications, therapy, changes in renal function, and discharge medications) will be compared between the SSU and usual care groups using the same methods as for baseline variables. A two-sided *p*-value less or equal to 0.05 is considered statistically significant (without multiplicity adjustment).

Comparison on DAOOH (primary outcome): The DAOOH will be compared between the two arms using two-sample t-test. Statistical significance will be declared at 5% level (two-sided). We will also fit a linear regression model, which includes the intervention arm indicator and the site variable (e.g. a categorical variable with four levels) as the covariates. The regression model will account for variation among sites so that the power in detecting intervention effect can be improved. It will also allow us to assess potential heterogeneity in intervention effect among sites through testing the interaction between the two covariates.

Capture of DAOOH Endpoint: Each study site uses an electronic health record with time stamps for ED arrival, time leaving the ED, time of arrival to SSU or inpatient admission, and time of discharge. Thus, exact times, rounded to the nearest hour, will be calculated to determine length of stay. Any time spent in the ED, SSU, or inpatient admission will count against their DAOOH. Although the endpoint uses the term "hospital," for the purposes of this analysis and its patient-centered focus, any time in these three hospital settings (ED, SSU, inpatient admission) will be counted. If a patient returns to the hospital or to the ER, the time spent (in hours) will be added to their total length of stay. This time does not need to be consecutive.

10.4.3 ANALYSIS OF THE SECONDARY ENDPOINT(S)

QoL: An AHF SSU strategy of care will lead to significant improvement in QoL scores at 30 days post-discharge compared with usual care. QoL will be assessed with the KCCQ.[59]

Sample Size Calculation: The standard deviation of 1-week KCCQ change after acute heart failure is 20.8.[73] Below are the numbers of subjects (both arms combined) required to detect various intervention effects with 80% power and type I error controlled at 0.05 (two-sided) in the table.

KCCQ: Sample size (both arms combined)	% of data attrition	Difference in KCCQ change
480	10%	5.3
360	33%	6.2
240	55%	7.6
120	78%	10.7

Similarly, the number of subjects (both arms combined) required to detect various effect size for caregiver burden (CB) with 80% power and type I error controlled at 0.05 (two-sided) is listed below. The effect size is defined as the difference in CB score divided by the standard deviation. It is a widely-used measure of treatment effect, which is used here because the standard deviation of CB in the target population if it has not been robustly studied. Because we changed CB scales, we will use established MCID for each established scale.

KCCQ QoL Analysis: The KCCQ will be compared between the two arms using the same method as in Aim 1. We will consider three ways of analysis with different outcomes based on KCCQ scores. First, we will exclude subjects who die without KCCQ in the analysis. Second, we will set KCCQ=0 for those who die without the 30-day KCCQ and include these people in the analysis. Third, we will create a composite binary endpoint of KCCQ<c or death, where c is a threshold. Chi-square test and logistic regression will be used to compare this outcome. We will select several relevant values for the threshold c and tabulate the results. The three analysis schemes allow us to understand how robust the comparison of KCCQ is with respect to different treatments of death.

Cost-Effectiveness Analysis:

To assess the effectiveness of short stay unit (SSU) management, we will analyze the patient outcomes as defined by cost, quality-of-life (QOL), medical utilization, and caregiver burden using regression analysis, and combine these results into a multifaceted incremental cost-effectiveness ratio (ICER).

$$ICER = \frac{C_{SSU} - C_H}{E_{SSU} - E_H}$$

Here, E_{SSU} and C_{SSU} are the effectiveness and cost, respectively, of the SSU, while the E_H and C_H are the effectiveness and cost of the hospital admission. The incremental cost effectiveness ratio allows us to estimate the additional cost associated with SSU treatment proportionate to the increased effectiveness of its patient outcome. We expect SSU treatment to dominate hospital admission, resulting in greater effectiveness at a lower cost.[1,2] Therefore, we expect ICER to be negative, reflecting the cost savings of implementing SSU treatment.

Our goal is to evaluate the cost-effectiveness of the SSU strategy from a societal perspective, as we will account for the costs of care from both the hospital/insurer perspective and patient point-of-view. As discussed in more detail below, our primary challenges lie in obtaining accurate and complete costs incurred by the patient outside of the hospital setting. Given this potential limitation, at minimum, our CEA evaluation will be from the hospital/payer perspective.

We will use an episodic approach to costing, which accounts for any cost shifting to a later point in time. This is the preferred approach for the Centers for Medicaid and Medicare services. Specifically, we will examine the total cost of patient care from the index ED visit (study enrollment) for a period of 30 days. In the context of the episode, we will analyze the incidence of readmission, total days of hospitalization, utilization of outpatient care, total cost, and caregiver burden.

The outcome variables of interest are cost, effectiveness, and other characteristics of participant experience. Costs will be defined by total billable hospital costs (Table 1) and patient costs. Hospital costs include payments by insurance, patients, and third parties. While this type of cost information is most accurate for our purposes, it is also the most difficult to obtain. Recognizing the phenomenon of 'upcharging,' we will obtain hospital charges from each site as this is the easier data to acquire.

Given the difficulty of obtaining costs and payments, we will standardize hospitalization and SSU costs using Medicare reimbursement rates (See Sensitivity Analysis Section below). Patient costs will include self-reported out-of-pocket costs and time costs using a modified version of a previously published survey. (see attached) One limitation of self-reported data will be potential mismeasurement due to recall bias. Should the patient-completed survey lack a sufficient response rate for outpatient analysis, we will limit our analysis to the hospital portion of the treatment. Effectiveness will be defined by SF-12 and SF-6D metrics of patient self-reported QOL. The SF-12 has been previously used to evaluate outcomes for HF patients.[3] Effectiveness in ICER computations is often measured using quality-adjusted life years (QALYs), defined as:

$$QALY = \sum_{t=t_0}^{Z} \delta^{t-t_0} q_t P_t$$

where P_t is the probability of surviving to each year t, q_t is the preference weight, and δ is the time-discounting factor. As the SF-12 does not allow direct assessment of QALYs, we will convert the score into a SF-6D preference based score using the six health dimension scores from the SF-12[4,5] to generate the preference weights and probabilities necessary to conduct the cost-utility analysis. Since the participant will be completing the SF-12 survey at the beginning and end of the episode, all models estimating the effectiveness will include the baseline score in the vector of participant characteristics. Aside from cost and effectiveness, we will analyze the incidence of re-admission, the utilization of outpatient care, and caregiver burden, as detailed in our statistical analysis plan. While the incidence of re-admission will be obtained from both the electronic health record and patient self-report, the outpatient and caregiver burden are self-reported.

For each outcome variable of interest (patient costs, hospital costs, total episode costs, SF-12 scores, SF-6D scores, QALYs, incidence of readmission, total days of hospitalization, utilization of outpatient care, nursing care, and caregiver burden) we will estimate a predictive model accounting for the heterogeneity among participants in the intervention and control groups.

$$Y_i = \alpha + \beta_1 SSU_i + \beta_2 D_i + \beta_3 R_i + \beta_4 D_i * R_i + \beta_5 D_i * SSU_i + \beta_6 R_i * SSU_i + \beta_7 D_i * R_i * SSU_i + \gamma X_i + \delta H_h + \kappa_h + \tau_t + \varepsilon_i$$

where, Y_i is the outcome of interest for participant *i* described in the following paragraph. SSU_i is an indicator if the participant was assigned to the SSU treatment, D_i are the total number of hospital inpatient days, R_i is an indicator for a readmission. The interaction terms D_i*R_i and $D_i*R_i*SSU_i$ differentiate between continuous inpatient days and inpatient days separated with a readmission, for hospital admission and SSU group respectively. The interaction terms D_i*SSU_i, and R_i*SSU_i capture the effect of SSU on inpatient days and readmission rates. X_i is a vector of participant characteristics (age, gender, insurance status, etc.), H_h is a vector of hospital characteristics, κ_h, τ_t are hospital, and year fixed effects, and ε_i is an independent and identically distributed error term. The effect of the SSU assignment on the outcome measure will be captured by coefficients β_1, β_5, β_6, β_7, unbiased and consistent estimators of the intervention effect because of the random assignment of participants.

When estimating the re-admission rate, we will use a probabilistic model such as:

$$\Pr(R_i) = F(\alpha + \beta_1 SSU_i + \beta_2 D_i + \beta_3 D_i * SSU_i + \gamma X_i + \delta H_h + \kappa_h + \tau_t + \varepsilon_i)$$

where the probability of readmission will be a function of intervention status, SSU_i, days of inpatient care, D_i, the interaction between these, D_i*SSU_i, participant characteristics, X_i, vector of hospital characteristics, H_h , and fixed effects, κ_h, τ_t, and ε_i is an independent and identically distributed error term. The choice of the functional form for this model will depend on the incidence of readmission in the data

Random assignment to SSU will be tested in pre-analysis by comparing the means of the intervention and control group characteristics using t-tests, and probability of being assigned to intervention group conditional of participant characteristics, particularly health status.

Sensitivity Analysis for CEA

To test the robustness of our estimates, in view of the assumptions made about cost, the choice of effectiveness measure, and the probability specification function for readmission, we will re-evaluate our results with alternative definitions.

To test the sensitivity of ICER to definition of cost, we will construct a projected cost measure based on CMS reimbursement for ICD-10 and CPT codes for hospitalization and SSU participants. The benefits and shortcomings of this approach are outlined in Table 1. Should the SF-12 lack sufficient variability between SSU and hospitalized participants, converting the measure to SF-6D will reduce the remaining heterogeneity, magnifying the ICER cost estimate. We will repeat ICER with SF-12 to test the significance in the loss of heterogeneity due to conversion.

Table 1 Hospital Costs Reports

	**Pros**	**Cons**
1 **Payments**<br>Payments received by hospitals from all sources	• Most accurate reflection of costs of care.<br>• Cost for all types of insurance<br>• Includes patient share of cost	• Hospitals reluctant to report<br>• Does not include uncompensated care
2 **Reimbursements from CMS**<br>Payments from CMS for Medicare and Medicaid patients	• Accurate reflection of costs of care.<br>• Projected to non-CMS patients.<br>• 70% of all ED visits have Medicare or Medicaid as primary payer.	• Obtain claims report from CMS (?)<br>• Less accurate for non-CMS patients.<br>• Does not include patient share or uncompensated care.
3 **Charges**<br>Charges presented by hospital for each procedure	• Procedure specific charges<br>• Accurate reflection of the charges presented to insurance or CMS<br>• Hospitals more likely to report<br>• Includes all types of insurance, including uncompensated care.	• Charges to cost ratio may differ by treatment type<br>• Not an accurate reflection of the cost of care.<br>• Does not include patient share.
4 **Projected costs**<br>By CPT and ICD-10 codes transformed	• Approximate measure of CMS reimbursement<br>• 70% of all ED visits have Medicare or Medicaid as primary payer	• Lost accuracy of variation in cost<br>• Does not include patient share<br>• Less accurate for non-CMS patients

through CMS reimbursement rates.		

When analyzing the re-admission rates, we will use a multinomial binary logistic function, estimating the odds ratio of any re-admission as a function of SSU status. We will also estimate the specification using a negative binomial generalized linear model with a log-link function, to analyze the effect of SSU on the number of re-admissions.

10.4.3.1 ADDITIONAL ANALYSES

Exploratory Analyses: As observation is not an admission, payers cover out of pocket costs differently.

Guideline Adherence: Adherence to HF guidelines at time of discharge in the SSU arm is equivalent or better than adherence in the usual care arm.

Sample Size Calculation: We assume that the adherence percentage is 85% in the control arm.[64] With 240 subjects in each arm, we will have 80% power to detect an absolute improvement of 8% in the SSU arm, where the type I error rate is controlled at 0.05 (two-sided). Adherence to HF-Guidelines will be assessed based on the established Get-With-The-Guidelines HF (GWTG-HF) metrics for eligible patients. These measures have been previously well established.[64,74]

HF Guideline Adherence Analysis: Fisher's exact test will be used to compare the percentage of adherence. Please see below regarding sensitivity analysis and missing data approaches.

CB Scale: Sample size (both arms combined)	% of data attrition	Effect size in CB scale
480	10%	0.25
360	33%	0.30
240	55%	0.37
120	78%	0.51

Caregiver Burden Analysis: This will be similar to the KCCQ analysis. See table for sample size considerations.

Sub-group analysis: The nature of the sub-group analysis is exploratory and will be used to generate hypotheses for future testing. We will study four pre-specified sub-groups: 1) older patients, defined as greater than 75 years old; 2) gender; 3) race and 4) insurance status (Medicaid, self-pay, Medicare, Commercial). The rationale for selecting these sub-groups is based either on known cardiovascular disparities or there is limited data regarding an SSU strategy in these specific subgroups.[46] The hypothesis: there will be no differences in outcomes, QoL, or guideline adherence between any of these groups. If the primary endpoint is achieved, we aim to demonstrate the AHF SSU strategy is broadly applicable across all subgroups. If there are differences, however, this may either demonstrate areas in need of further study, or initial subgroups to target. To test these hypotheses, we will compare the treatment benefit between sub-groups by formal two-sample testing procedures using normal approximation (e.g. Z test). A permutation test will also be performed as a sensitivity analysis. Given the limited sample size and the exploratory nature of the sub-group analysis, no multiple comparison adjustment will be made for these tests.

Intervention Monitoring: To ensure fidelity of each component of the intervention between study arms, we will also measure the following individual components of the intervention throughout the trial at both the site and overall study level: 1) Baseline patient characteristics, 2) Medicine reconciliation 3) Guideline-HF adherence 4) SSU AHF protocol adherence, 5) admission rates from the SSU 6) Quality of Life/Caregiver Burden Questionnaire response and completeness, 7) Hospitalized patients discharged within 48 hours, and 8) Follow up completed at 30 days. These analyses will be reported every 3 months to the Steering Committee to allow for course correction as needed. In addition, these will be reported at the conclusion of the study as well as at quarterly enrollment milestones to the DSMB.

Sensitivity Analyses: Some baseline covariates such as co-morbidities, medications, renal function, serum sodium, potassium, b-type natriuretic peptide (BNP) or NT-pro BNP, and blood pressure, may not be well-balanced between the two arms despite randomization. These covariates are known markers of risk in AHF patients and are part of the standard assessment for the vast majority of AHF admissions. We will use the inverse probability weighting approach to adjust those covariates with poor balance between the arms as part of a sensitivity analysis.[75] We will then compare these results with those from the two-sample t test. In addition, if the DAOOH, QoL, caregiver burden, or mRUQ-HF endpoints are found to be non-normal in distribution, the Wilcoxon Rank-Sum test will be used for comparison as a sensitivity analysis.

Modified Resource Utilization Questionnaire for Heart Failure (mRUQ-HF).[70] The mRUQ-HF is a14-item self-report questionnaire of comprehensive lists of choice related to healthcare utilization. (See Appendix trial protocol) Exploratory analysis will occur in two steps: 1) Determination of resources consumed or related; 2) Assignment of 'value' to the resources consumed.[71] This two-step process allows for transparency of assigned costs. Hospital costs will be assigned per individual hospital cost-accounting. Where monetary values are not directly self-reported, Medicare fee schedules will be used for uniformity. This will be captured at 90 days and will include the hospitalization when patients answer the questionnaire.

All Cause Mortality and All-Cause Rehospitalization Endpoints:

A composite endpoint of all-cause mortality and re-hospitalization is defined as the time from the randomization to either all-cause death, or re-hospitalization. The two intervention arms will be compared using a log-rank test. Given the multiple hospitals, a stratified log rank test will be utilized.

To compare the proportions of patients who had either all cause mortality or re-hosptialization within 30 days and separately 90 days from randomization, a composite endpoint of all-cause mortality and re-hospitalization is defined as either 1 for patients who died or re-hospitalized with 90 days since randomization, or 0 otherwise. The two intervention arms will be compared using the Fisher's exact test or Chi-square test as appropriate.

10.4.4 SAFETY ANALYSES

As all cause mortality and re-hospitalizations will already be reported as part of the efficacy exploratory analyses, these will also be highlighted as safety analyses. We will also report the proportion of patients who are admitted from the SSU as well as AE/SAE's.

10.4.5 ADHERENCE AND RETENTION ANALYSES

Missing data: This study does not anticipate significant missing data because the follow-up is fairly short. However, we have planned for this unlikely possibility in both our sample size calculations and analysis plan. The magnitude of any missing data and their relevant distributions will be properly documented. Multiple-imputation[42] will be used to analyze the incomplete data under the assumption of missing at random (MAR).[43] If it is of concern that the data may be missing not at random (MNAR),[43] then a sensitivity analysis will be performed under multiple imputation scheme.[44] Summary data at baseline based on subjects with legitimate values on corresponding variables will be reported. For main inferential statistics, results based on the strategy of ignoring the missing data and multiple imputations will be reported so that the impact of missing data can be assessed.

To ensure completeness, medical records will also be reviewed for items such as past medical history, lab results, medications, and other test results. The DCC will perform data audits at periodic intervals to assess the integrity of the data, also checking for missing data. Any fields with more than 5% missing data will be flagged for further investigation, with queries to sites to ascertain the reasons and/or fix accordingly. If needed, protocols or procedures will be implemented, along with further education, to minimize further missing data. In terms of drop-outs, a well-trained study team works to minimize this through careful screening and informed consent. While patients may withdraw their consent at any time, reasons for withdrawal will be queried and tabulated. For patients who drop out, the reason for dropping out will be queried and recorded. These will be tabulated to determine if specific reasons for drop-outs are occurring, which will then be addressed by the Executive Committee. Every attempt to complete assessments for the

outcomes of interest to this study will be undertaken. In keeping with the pragmatic, simple nature of this trial design, there will be no clinical events or adjudication committee, as total DAAOH will be counted, irrespective of mode of death or re-hospitalization.

10.4.6 Baseline Descriptive Statistics

Patient baseline characteristics will be tabulated for both SSU and usual care groups. Specifically, means (standard deviations) or medians (interquartile range) will be presented for continuous variables such as age and body mass index. For categorical variables, such as gender and race, frequency and percentages will be presented. Group comparisons of continuous variables will be based on either two-sample t-tests (under normality) or Wilcoxon's Rank Sum test. Normality of distribution will be determined using the Kolmogorov-Smirnov goodness-of-fit test. Categorical data comparisons will be presented based on the Chi-square or Fisher's exact test. Standardized difference is used to compare the mean of the SSU and usual care groups.[72]

10.4.7 PLANNED INTERIM ANALYSES

Formal interim analysis will occur arfter 50% of patients have been accrued. NO formal stopping rules have been established, given the clinical effectiveness study design. IN other words, safety concerns are unlikely and equivalent outcomes would not be defined as futile. Rather, the DSMB will issue recommendations on halting the trial early, only for safety reasons, based on the totality of data reviewed.

10.5 SAMPLE SIZE

A one-day difference in the length of hospital stay is a 20% reduction in the mean length of stay(LOS) of five days during hospitalization (median LOS =4.3 days).[50] Pilot data support the clinical significance of a one-day reduction,[45] and discussions with patients and caregivers confirms this finding. *While we anticipate achieving greater than a one-day difference, we have powered our study conservatively.*

N (total) = 534; **N_1** = 267; **N_2** = 267

Based on published data,[46] we project that DAOOH has a mean of 25.6 with a standard deviation of 3.9 in the control arm. A reduction of the mean DAOOH by one-day is the minimal clinically meaningful difference as determined through our patient engagement outreach.[54] An interim analysis when half of the outcome data are available will be performed. (See DSMB, section E6). Accounting for this with the O'Brien-Fleming spending function, 240 subjects per arm are required to achieve 80% power in detecting the one-day difference in DAOOH, where the two-sided type I error rate is controlled at 0.05. Assuming 10% attrition due to drop-outs and lost to follow up, this study requires a total of 534 subjects. This power calculation also takes into account a conservative estimate of 40% of patients being admitted to the hospital from the SSU arm. *DAOOH automatically accounts for death*. The maximal number of days alive and out of the hospital through 30 days post randomization is 30 days. If a patient were to die on day 8 post-randomization, her DAOOH would be 8. This would be compared to a patient who stayed in the hospital 3 days after randomization, but never returned to the ED or hospital or died through 30 days. DAAOH of this patient would be 27. If the same patient returned to the hospital on day 12 for 2 days, and again on day 24 for 2 days, their total DAOOH would be 23.

10.6 MEASURES TO MINIMIZE BIAS

10.6.1 ENROLLMENT/ RANDOMIZATION/ MASKING PROCEDURES

Patients will be randomized 1:1 to one of the two study arms, but stratified by site to ensure equal site representation. A central computer generated randomization scheme with random block sizes of two, four, and six will be created, stratified by site. The REDCap randomization module will be utilized to generate the randomization schema and patient allocation.

Due the nature of the intervention and the clinical setting, this is an unblinded trial.

11 SOURCE DOCUMENTS AND ACCESS TO SOURCE DATA/DOCUMENTS

The medical experts, study monitors, auditors, and health authority inspectors (or their agents) will be given direct access to source data and documentation (e.g., medical charts/records, laboratory test results, printouts, videotapes) for source data verification, provided that patient confidentiality is maintained in accordance with local requirements.

Each Investigator must maintain, at all times, the primary records (i.e., source documents) of each patient's data. Examples of source documents are hospital records, office visit records; examining physician's finding or notes, consultant's written opinion or notes, laboratory reports, drug inventory, study drug label records, and CRFs that are used as the source.

Each Investigator will maintain a confidential patient identification list that allows the unambiguous identification of each patient. All study-related documents must be kept for a minimum of 5 years. A publically available dataset will be released per NIH guidelines.

12 QUALITY ASSURANCE AND QUALITY CONTROL

Protocol Amendments: No changes from the final approved (signed) protocol will be initiated without the prior written approval or favorable opinion of a written amendment by the IEC/IRB, except when necessary to eliminate immediate safety concerns to the patients or when the change involves only logistics or administration. Each Investigator will sign the protocol amendment.

The IRB/EC may provide expedited review and approval/favorable opinion for minor change(s) in ongoing studies.

Protocol Deviations, Violations, and Exceptions: A **protocol deviation** is non-adherence to protocol-specific study procedures or schedules that does not involve inclusion/exclusion criteria, primary objective variable criteria, and/or GCP guidelines. Deviations are considered minor and do not impact the study.

A **protocol violation** is any significant divergence from the protocol, i.e., non-adherence on the part of the patient, the Investigator, or the sponsor to protocol-specific inclusion/exclusion criteria, primary objective variable criteria, and/or GCP guidelines. Protocol violations will be identified and recorded, by study center personnel.

No **exceptions** to protocol-specific entry criteria will be granted to allow patients to enter a study.

Information to Study Personnel: Each Investigator is responsible for giving information about the study to all staff members involved in the study or in any element of patient management, both before starting the practical performance of the study and during the course of the study (e.g., when new staff become involved). Each Investigator must assure that all study staff members are qualified by education, experience, and training to perform their specific responsibilities. These study staff members must be listed on the study center authorization form, (if required) which includes a clear description of each staff member's responsibilities. This list must be updated throughout the study, as necessary.

The study monitor is responsible for explaining the protocol to all study staff, including each Investigator, and for ensuring their compliance with the protocol. Additional information will be made available during the study when new staff become involved in the study and as otherwise agreed upon with either the Investigator or the study monitor.

The handling of data, including data quality assurance, will comply with regulatory guidelines (e.g., ICH and GCP) and the sponsor's or its designee's SOPs and working instructions. Data management and control processes specific to this study will be described in a data management plan. When data management is outsourced, the contract organization will be responsible for the development and implementation of the data management plan.

Data Quality Assurance: All data on the CRF will be entered into a validated database compliant with 21 CFR Part 11 requirements. In the case when data management is outsourced, the contract organization will be responsible for database quality assurance including, but not limited to, review of data entered into the CRFs by study center personnel for completeness and accuracy and instruction of the study personnel to make any required corrections.

Data management at Indiana University will implement edit checks on the eCRF to enforce data integrity and compliance to the protocol and regulatory requirements. Study center personnel will be responsible for entering study data on the eCRFs. Data management will track eCRFs and review them for completeness, the presence of mandatory values, consistency, and dated electronic signatures. Queries identified during data discrepancy review will be sent to the study center personnel to be reviewed and resolved in a timely manner.

Adverse Events will be coded using the MedDRA dictionary. Concomitant medications will be coded using the WHO Drug dictionary. Adverse Events and Concomitant Medications will be reviewed for coding consistency and completeness.

At the end of the study, the database will be locked and the data will be released for reporting and statistical analysis.

13 ETHICS/PROTECTION OF HUMAN SUBJECTS

13.1 ETHICAL STANDARD

The Investigator(s) will conduct the study in accordance with this protocol, the guiding principles of the Declaration of Helsinki, ICH GCP guidelines and applicable regulatory requirements.

13.2 INSTITUTIONAL REVIEW BOARD

Before this study starts, the protocol will be submitted to each IEC/IRB for review. As required, the study will not start at a given center before the IEC/IRB for the center provides written approval or a favorable opinion. The IRB will meet all FDA requirements governing IRBs (Code of Federal Regulations, Title 21, Part 56). The IEC will meet local regulations.

13.3 INFORMED CONSENT PROCESS

13.3.1 CONSENT/ASSENT AND OTHER INFORMATIONAL DOCUMENTS PROVIDED TO PARTICIPANTS

Each patient must be provided with a statement that the investigation involves research and that the IRB/EC has approved solicitation of patients to participate; a fair explanation of the procedures to be followed and their purposes, including identification of any procedures which are experimental; a description in lay language of any possible side effects; a description of any attendant discomforts and risks reasonably to be expected; a description of any benefits reasonably to be expected; a disclosure of any appropriate alternative procedures that might be advantageous for the patient; an offer to answer any inquiries concerning the procedures, and instruction that the person is free to withdraw consent and discontinue participation in the project or activity at any time without prejudice to the patient. The informed consent shall include a disclosure that the Investigator is being supported by the NIH to perform the stated research.

13.3.2 CONSENT PROCEDURES AND DOCUMENTATION

A properly executed, written consent in compliance with current U.S. federal code 21CFR part 50, or competent regulatory authority, shall be obtained from each patient prior to entering the study or prior to performing any unusual or non-routine procedure involving risk to the patient.

A patient must give written consent to participate in the study. This consent must be dated and retained by the Principal Investigator as part of the study records. A copy shall be given to the patient. The informed consent process must be documented in the patient's source documents.

Written and/or oral information about the study in a language understandable by the patient will be given to all patients.

13.4 PARTICIPANT AND DATA CONFIDENTIALITY

Each Investigator must assure that the privacy and confidentiality of each study patient's, personal identity and personal medical information is, maintained at all times. In order to maintain subject privacy and confidentiality, all CRFs, laboratory specimens, evaluation forms, reports, and other records, documents and image material that leave the site will be identified only by an identification code. This identification code shall on no occasion include study subject's names, initials or date of birth.

Personal medical information may release or review the personal health data of study patients shall take place solely within circumstance, and to third parties, specifically identified by the written informed consent document signed by the study patients, except as permitted by applicable laws and regulations for purposes of monitoring and data verification by the relevant regulatory authorities, the NIH and NIH's properly authorized representatives, the quality assurance unit, or regulatory authorities. Personal medical information will always be treated as confidential.

14 DATA HANDLING AND RECORD KEEPING

14.1 DATA COLLECTION AND MANAGEMENT RESPONSIBILITIES

Each Investigator must keep a separate patient identification list showing code numbers, names, and dates of birth to allow unambiguous identification of each patient included in the study. A note will be made in the medical records that the patient is participating in a clinical study.

All required data will be recorded on the CRF by study center personnel according to the data entry guidelines provided by the PI or designee. All CRFs must be kept in good order and updated so they always reflect the latest observations on the patients participating in the study.

When paper CRFs are used, they will be completed legibly in black ink, with reasons given for missing data. Any corrections to the data will be made in a manner that does not obscure the original entry and will be dated and initialed by the Investigator or assigned designee. Each Investigator will sign the statement on the last page of the CRF.

When eCRFs are used, electronic signatures of the Investigator (or designee) will be provided.

Access to the eCRF for data entry and signature is controlled by user identification and password, which are provided by the PI or designee. Study center personnel will be trained, by the PI or designee, in the use of eCRFs and application of electronic signatures before the start of the study.

Because it is extremely important to have proper data collection in a timely manner, the Investigator shall complete the CRFs and on an ongoing basis. If a study monitor is needed and study monitor requests additional data or clarification of data for the CRF, the request must be answered satisfactorily in a timely manner before the next monitoring visit.

14.2 STUDY RECORDS RETENTION

All records related to the study (i.e., source data, source documents, CRFs, copies of protocols and protocol amendments, correspondence, patient identification lists, signed informed consent forms, and other essential documents) must be retained for a minimum of 5 years.

Should an Investigator wish to assign the study records to another party or move them to another location, advance written notice will be given to the PI and NIH.

The Investigator will maintain all study records according to International Conference on Harmonization (ICH)-GCP and applicable regulatory requirements. Records will be retained for two (2) years following the date a marketing application is approved for the indication pertaining to this clinical study; or, if the medication is planned to be terminated or if a Regulatory application is not planned to be progressed, until two (2) years after the investigation is discontinued and the Food and Drug Administration (FDA), or competent regulatory authority, is notified.

14.3 PROTOCOL DEVIATIONS

A **protocol deviation** is non-adherence to protocol-specific study procedures or schedules that does not involve inclusion/exclusion criteria, primary objective variable criteria, and/or GCP guidelines. Deviations are considered minor and do not impact the study.

A **protocol violation** is any significant divergence from the protocol, i.e., non-adherence on the part of the patient, the Investigator, or the sponsor to protocol-specific inclusion/exclusion criteria, primary objective variable criteria, and/or GCP guidelines. Protocol violations will be identified and recorded, by study center personnel.

No **exceptions** to protocol-specific entry criteria will be granted to allow patients to enter a study.

14.4 PUBLICATION AND DATA SHARING POLICY

RESOURCE SHARING, DISSEMINATION, AND DATA SHARING PLAN

This includes the following activities:

- Publicizing the study as it is initiated, to trial investigators and other interested researchers
- Identifying and supporting proposals, (funding sources, implementation, analyses) for feasible salient ancillary studies;
- Providing a fully anonymized data set for future analyses/studies by interested researchers, once the trial funding has ended.

Each of these activities is discussed below.

Publicizing the Study

The key methods for publicizing the trial are:

- ClinicalTrials.gov – the government website that registers all initiated trials with trial protocol descriptions and contact information;
- The trial site PI's and involved leadership group
- Design and rationale paper will be submitted for publication

Providing Access to Linked, Anonymized Data

Once trial funding ends, two options will be:

- Provide the anonymized data set to AHRQ, if desired
- Maintain the linked, anonymized data set via the IU Data Coordinating Center.

Regardless of which option is implemented, information on availability of data will be accessible on ClinicalTrials.gov and will be linked to other appropriate sites, including Indiana University websites.

PREPARATION OF THE ANONYMIZED, LIMITED ACCESS DATASET

We propose the following activities for the data set:

1. Subject identifiers:
    a. New random identification numbers without site identifiers will replace the original identification numbers, once data acquisition is complete.
    b. The key linking the original and new ID numbers will NOT be provided to users of the anonymized data set, nor to site investigators and staff.
2. Variables that might lead to the identification of participants:
    a. Interviewer or technician identification numbers or codes will be recoded or deleted.
    b. Regional variables with little or no variation within a center because they could be used to identify the center will be deleted.
    c. Unedited, verbatim responses that are stored as text data (e.g., specified in “other” category) will be deleted.
3. Dates: All dates will be coded relative to a specific reference point (e.g., date of randomization). This provides privacy protection for individuals known to be in a study who are known to have had some significant event (e.g., myocardial infarction) on a particular date. Birth and other milestone dates will also be recoded relative to a specific reference date.
4. Variables with low frequencies for some values, that might be used to identify participants, may be recoded. These might include:
    a. Socioeconomic and demographic data (e.g., marital status, occupation, income, education, language, number of years married).
    b. Household and family composition (e.g., number in household, number of siblings or children, ages of children or step-children, number of brothers and sisters, relationships, spouse in study).
    c. Numbers of pregnancies, births, or multiple children within a birth.
    d. Anthropometry measures (e.g., height, weight, waist girth, hip girth, body mass index).
    e. Physical characteristics that are distinctive (e.g., blindness).
    f. Prior medical conditions with low frequency (e.g., group specific cancers into broader categories) and related questions such as age at diagnosis and current status.
5. Race/ethnicity and gender information when very few subjects are in certain groups or cells.

a. Polychotomous variables: values or groups will be collapsed so as to ensure a minimum number of subjects (e.g., at least 20) for each value within each race-gender cell.
b. Continuous variables: distributions will be truncated if needed to ensure that a minimum number of subjects (e.g., at least 20) have the same highest and lowest values in each race-gender cell.
c. Dichotomous variables: data should either be grouped with other related variables so as to ensure a minimum number of subjects (e.g., at least 20) in each race-gender cell or deleted.

This includes the following activities:

- Publicizing the study as it is initiated, to trial investigators and other interested researchers
- Identifying and supporting proposals, (funding sources, implementation, analyses) for feasible salient ancillary studies;
- Providing a fully anonymized data set for future analyses/studies by interested researchers, once the trial funding has ended.

Each of these activities is discussed below.

Publicizing the Study

The key methods for publicizing the trial are:

- ClinicalTrials.gov – the government website that registers all initiated trials with trial protocol descriptions and contact information;
- The trial site PI's and involved leadership group
- Design and rationale paper will be submitted for publication
- A final manuscript will be submitted after conclusion of the study.

Providing Access to Linked, Anonymized Data

Once trial funding ends, two options will be:

- Provide the anonymized data set to AHRQ.
- Maintain the linked, anonymized data set via the IU Data Coordinating Center.

Regardless of which option is implemented, information on availability of data will be accessible on ClinicalTrials.gov and will be linked to other appropriate sites, including Indiana University websites.

PREPARATION OF THE ANONYMIZED, LIMITED ACCESS DATASET

To comply with NIH requirements for an adequately anonymized dataset, we propose the following activities for the data set:

6. Subject identifiers:
    a. New random identification numbers without site identifiers will replace the original identification numbers, once data acquisition is complete.
    b. The key linking the original and new ID numbers will NOT be provided to users of the anonymized data set, nor to site investigators and staff.
7. Variables that might lead to the identification of participants:
    a. Interviewer or technician identification numbers or codes will be recoded or deleted.
    b. Regional variables with little or no variation within a center because they could be used to identify the center will be deleted.
    c. Unedited, verbatim responses that are stored as text data (e.g., specified in “other” category) will be deleted.
8. Dates: All dates will be coded relative to a specific reference point (e.g., date of randomization). This provides privacy protection for individuals known to be in a study who are known to have had some significant event

(e.g., myocardial infarction) on a particular date. Birth and other milestone dates will also be recoded relative to a specific reference date.

9. Variables with low frequencies for some values, that might be used to identify participants, may be recoded. These might include:
    a. Socioeconomic and demographic data (e.g., marital status, occupation, income, education, language, number of years married).
    b. Household and family composition (e.g., number in household, number of siblings or children, ages of children or step-children, number of brothers and sisters, relationships, spouse in study).
    c. Numbers of pregnancies, births, or multiple children within a birth.
    d. Anthropometry measures (e.g., height, weight, waist girth, hip girth, body mass index).
    e. Physical characteristics that are distinctive (e.g., blindness).
    f. Prior medical conditions with low frequency (e.g., group specific cancers into broader categories) and related questions such as age at diagnosis and current status.
10. Race/ethnicity and gender information when very few subjects are in certain groups or cells.
    a. Polychotomous variables: values or groups will be collapsed so as to ensure a minimum number of subjects (e.g., at least 20) for each value within each race-gender cell.
    b. Continuous variables: distributions will be truncated if needed to ensure that a minimum number of subjects (e.g., at least 20) have the same highest and lowest values in each race-gender cell.
    c. Dichotomous variables: data should either be grouped with other related variables so as to ensure a minimum number of subjects (e.g., at least 20) in each race-gender cell or deleted.

CLINICAL TRIALS.GOV

This study will be registered at the appropriate and required time by the PI, in conjunction with the DCC, to the government-operated clinical trial registry data bank, which contains registration, results, and other information about registered clinical trials at ClinicalTrials.gov. Federal law under FDAAA requires clinical trial information for certain clinical trials to be submitted to the data bank and this study will comply with all reporting requirements for clinical trials.

15 STUDY ADMINISTRATION

15.1 STUDY LEADERSHIP

This study will be conducted at 3 sites in the United States, with 4 total hospitals. Site 1) Indianapolis, IN at both the Eskenazi and Methodist hospitals (abbreviated as IU, Peter S. Pang, PI), Site 2) Nashville, TN at the University of Vanderbilt hospital (abbreviated as Vanderbilt, Sean P. Collins, PI), Site 3) Detroit, MI at the Detroit Receiving Hospital Center (abbreviated as DRC, Philip D. Levy. Each site PI will be a member of the steering committee. Dr. Susan Pressler will also be a member of the steering committee. Dr. Sean Collins will chair the steering committee.

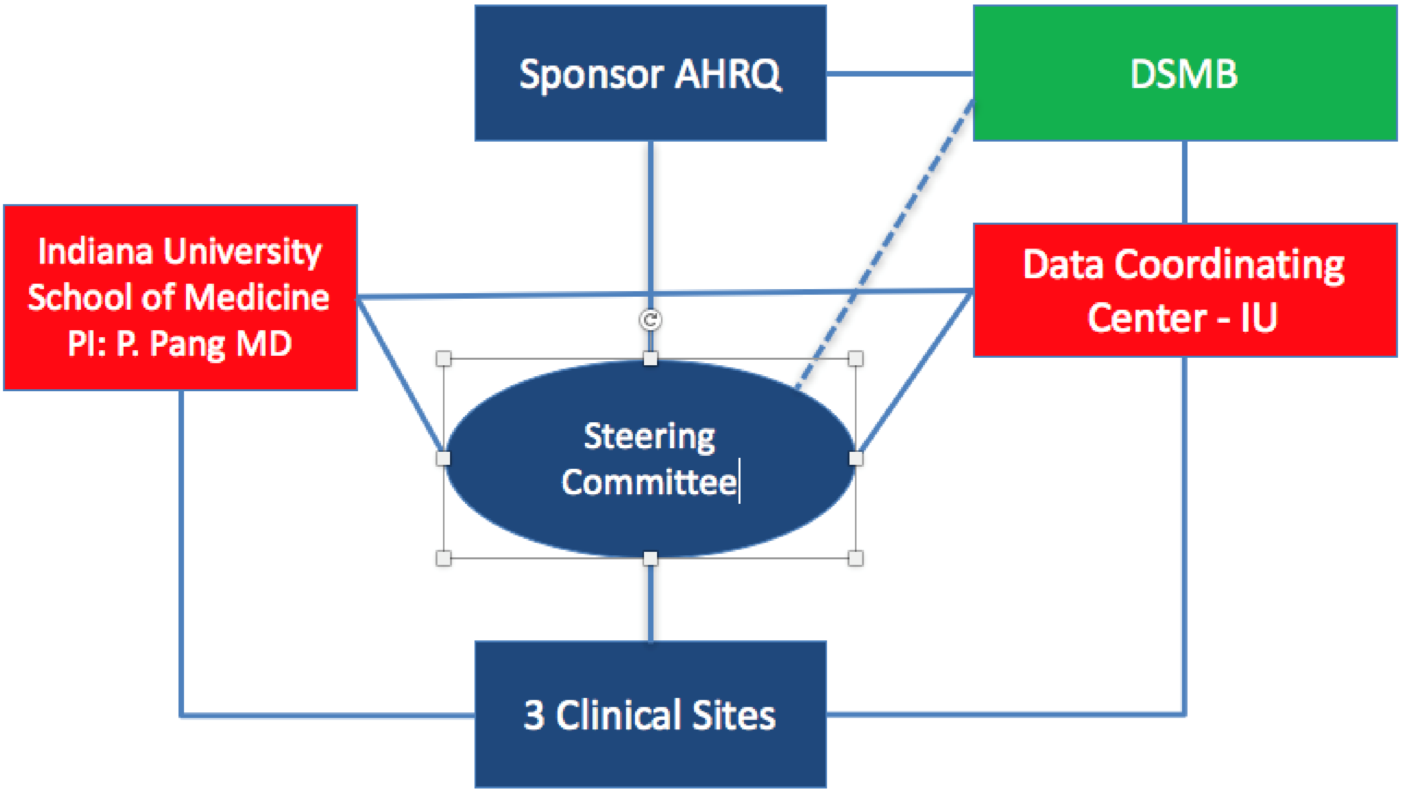

Communication with DSMB members will be primarily through the AHRQ Program Office and the Data Coordinating Center (DCC) housed at IU. The primary coordinator of data transfer between the research team and the DSMB will be the DCC working with the DSMB statistician, who will also be at IU. It is expected that study investigators will not communicate with DSMB members about the study directly, except when making presentations or responding to questions at DSMB meetings or during conference calls.

16 CONFLICT OF INTEREST POLICY

All investigators must adhere to national, regional, and local conflict of interest policies. Prior to publication, all disclosures potentially relevant to this trial will be explicitly stated.

17 SURVEYS AND QUESTIONNAIRES

17.1 KCCQ

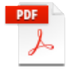

New KCCQ.pdf

17.2 CAREGIVER BURDEN SCALES

OBERST CAREGIVING BURDEN SCALE

This group of questions is about the tasks and activities that you do to help the patient at home. For each of the following activities, please mark how much **time you spend** and **how difficult** each activity is for you to do.

1. Medical or nursing treatments (giving medications, skin care, dressings, etc.):

**Time you spend:**	**How difficult:**
___ A great amount (5)	___ Extremely difficult (5)
___ A large amount (4)	___ Very difficult (4)
___ A moderate amount (3)	___ Moderately difficult (3)
___ A small amount (2)	___ Slightly difficult (2)
___ None (1)	___ Not difficult (1)

___About how many hours/day

___About how many hours/week

2. Personal care (bathing, toileting, getting dressed, feeding, etc.):

**Time you spend:**	**How difficult:**
___ A great amount (5)	___ Extremely difficult (5)
___ A large amount (4)	___ Very difficult (4)
___ A moderate amount (3)	___ Moderately difficult (3)
___ A small amount (2)	___ Slightly difficult (2)
___ None (1)	___ Not difficult (1)

___About how many hours/day

___About how many hours/week

3. Managing dietary needs of the patient (planning and cooking meals, monitoring salt intake, etc.):

**Time you spend:**	**How difficult:**
___ A great amount (5)	___ Extremely difficult (5)
___ A large amount (4)	___ Very difficult (4)
___ A moderate amount (3)	___ Moderately difficult (3)

___ A small amount (2) ___ Slightly difficult (2)

___ None (1) ___ Not difficult (1)

___About how many hours/day

___About how many hours/week

4. Assistance with walking, getting in and out of bed, exercises, etc.:

SPEND:

**Time you spend:**	**How difficult:**
___ A great amount (5)	___ Extremely difficult (5)
___ A large amount (4)	___ Very difficult (4)
___ A moderate amount (3)	___ Moderately difficult (3)
___ A small amount (2)	___ Slightly difficult (2)
___ None (1)	___ Not difficult (1)

___About how many hours/day

___About how many hours/week

5. Emotional support, "being there" for the patient:

TIME YOU SPEND: HOW DIFFICULT:

**Time you spend:**	**How difficult:**
___ A great amount (5)	___ Extremely difficult (5)
___ A large amount (4)	___ Very difficult (4)
___ A moderate amount (3)	___ Moderately difficult (3)
___ A small amount (2)	___ Slightly difficult (2)
___ None (1)	___ Not difficult (1)

___About how many hours/day

___About how many hours/week

6. Watching for and reporting the patient’s symptoms, watching how the patient is doing, monitoring the patient’s progress:

TIME YOU SPEND: HOW DIFFICULT:

**Time you spend:**	**How difficult:**
___ A great amount (5)	___ Extremely difficult (5)
___ A large amount (4)	___ Very difficult (4)
___ A moderate amount (3)	___ Moderately difficult (3)
___ A small amount (2)	___ Slightly difficult (2)
___ None (1)	___ Not difficult (1)

___About how many hours/day

___About how many hours/week

7. Providing transportation or “company” (driving, riding along with patient, going to appointments, driving patient around for errands, etc.):

**Time you spend:**	**How difficult:**
___ A great amount (5)	___ Extremely difficult (5)
___ A large amount (4)	___ Very difficult (4)
___ A moderate amount (3)	___ Moderately difficult (3)
___ A small amount (2)	___ Slightly difficult (2)
___ None (1)	___ Not difficult (1)

___About how many hours/day

___About how many hours/week

8. Managing finances, bills, and forms related to the patient’s illness:

**Time you spend:**	**How difficult:**
___ A great amount (5)	___ Extremely difficult (5)
___ A large amount (4)	___ Very difficult (4)
___ A moderate amount (3)	___ Moderately difficult (3)
___ A small amount (2)	___ Slightly difficult (2)

___ None (1) | ___ Not difficult (1)

___About how many hours/day

___About how many hours/week

9. Additional household tasks for the patient (laundry, cooking, cleaning, yard work, home repairs, etc.):

TIME YOU SPEND: HOW DIFFICULT:

**Time you spend:**	**How difficult:**
___ A great amount (5)	___ Extremely difficult (5)
___ A large amount (4)	___ Very difficult (4)
___ A moderate amount (3)	___ Moderately difficult (3)
___ A small amount (2)	___ Slightly difficult (2)
___ None (1)	___ Not difficult (1)

___About how many hours/day

___About how many hours/week

Additional tasks outside the home for the patient (shopping for food and clothes, going to the bank, running errands, etc.):

**Time you spend:**	**How difficult:**
___ A great amount (5)	Extremely difficult (5)
___ A large amount (4)	___ Very difficult (4)
___ A moderate amount (3)	___ Moderately difficult (3)
___ A small amount (2)	___ Slightly difficult (2)
___ None (1)	___ Not difficult (1)

___About how many hours/day

___About how many hours/week

10. Structuring/planning activities for the patient (recreation, rest, meals, things for the patient to do, etc.):

TIME YOU SPEND: HOW DIFFICULT:

**Time you spend:**	**How difficult:**
___ A great amount (5)	___ Extremely difficult (5)
___ A large amount (4)	___ Very difficult (4)
___ A moderate amount (3)	___ Moderately difficult (3)
___ A small amount (2)	___ Slightly difficult (2)
___ None (1)	___ Not difficult (1)

___About how many hours/day

___About how many hours/week

11. Managing behavior problems in terms of the patient's moodiness and irritability:

TIME YOU SPEND: HOW DIFFICULT:

**Time you spend:**	**How difficult:**
___ A great amount (5)	___ Extremely difficult (5)
___ A large amount (4)	___ Very difficult (4)
___ A moderate amount (3)	___ Moderately difficult (3)
___ A small amount (2)	___ Slightly difficult (2)
___ None (1)	___ Not difficult (1)

___About how many hours/day

___About how many hours/week

12. Managing behavior problems in terms of the patient's memory loss, concentration, and attention:

**Time you spend:**	**icult:**
___ A great amount (5)	___ Extremely difficult (5)
___ A large amount (4)	___ Very difficult (4)
___ A moderate amount (3)	___ Moderately difficult (3)
___ A small amount (2)	___ Slightly difficult (2)

___ None (1) ___ Not difficult (1)

___About how many hours/day

___About how many hours/week

13. Managing behavior problems in terms of the patient's confusion, disorientation, or dementia:

**Time you spend:**	**How difficult:**
___ A great amount (5)	___ Extremely difficult (5)
___ A large amount (4)	___ Very difficult (4)
___ A moderate amount (3)	___ Moderately difficult (3)
___ A small amount (2)	___ Slightly difficult (2)
___ None (1)	___ Not difficult (1)

___About how many hours/day

___About how many hours/week

14. Finding and arranging someone to care for the patient while you are away:

**Time you spend:**	**How difficult:**
___ A great amount (5)	___ Extremely difficult (5)
___ A large amount (4)	___ Very difficult (4)
___ A moderate amount (3)	___ Moderately difficult (3)
___ A small amount (2)	___ Slightly difficult (2)
___ None (1)	___ Not difficult (1)

___About how many hours/day

___About how many hours/week

Communication (helping the patient with the phone, writing or reading, explaining things, etc):

**Time you spend:**	**How difficult:**
___ A great amount (5)	[illegible]ely difficult (5)
___ A large amount (4)	___ Very difficult (4)
___ A moderate amount (3)	___ Moderately difficult (3)
___ A small amount (2)	___ Slightly difficult (2)
___ None (1)	___ Not difficult (1)

___About how many hours/day

___About how many hours/week

15. Coordinating, arranging, and managing services and resources for the patient (scheduling appointments, arranging transportation, locating equipment and services, and finding outside help):

**Time you spend:**	**How difficult:**
___ A great amount (5)	___ Extremely difficult (5)
___ A large amount (4)	___ Very difficult (4)
___ A moderate amount (3)	___ Moderately difficult (3)
___ A small amount (2)	___ Slightly difficult (2)
___ None (1)	___ Not difficult (1)

___About how many hours/day

___About how many hours/week

16. Seeking information and talking with doctors, nurses, and other professional health care workers about the patient’s condition and treatment plans:

Time you spend:	How difficult:
___ A great amount (5)	___ Extremely difficult (5)
___ A large amount (4)	___ Very difficult (4)
___ A moderate amount (3)	___ Moderately difficult (3)
___ A small amount (2)	___ Slightly difficult (2)
___ None (1)	___ Not difficult (1)

___About how many hours/day

___About how many hours/week

Bakas Caregiving Outcomes Scale (BCOS)

Site Specific ID or MRN

Page 1 of 2

Was a caregiver identified? Defined as person either self-identifies, or when asked identifies themselves, as the primary caregiver for the patient.	No<br>Yes
Person who filled out form	Caregiver filled out the form<br>Research staff Asked the caregiver in person<br>Research staff Asked thecaregiver by phone

This group of questions is about the possible changes in your life from providing care for your family member or loved one. For each possible change listed, circle one number indicating the degree of change. The numbers indicating the degree of change range from 3 Changed for the Worst to +3 Changed for the Best. The number 0 means Did Not Change.

1. My self esteem
2. My physical health
3. My time for family activities
4. My ability to cope with stress
5. My relationship with friends
6. My future outlook
7. My level of energy

8. My emotional well-being

9. My roles in life

10. My time for social activities with friends

-3 Changed for the Worst

-2 -1 0 Did Not Change

1 2 3 Changed for the Best

11. My relationship with my family

12. My financial well-being

13. My relationship with the heart failure patient

14. My physical functioning

15. My general health

16. In general, how has your life changed as a result of taking care of the heart failure patient?

If there any other changes in your life as a result of providing care, please write them below and rate them accordingly.

17 Description

17 Rating
-3 Changed for the Worst
-2
-1
0 Did Not Change 1
2
3 Changed for the Best

18 Description

18 Rating
-3 Changed for the Worst
-2
-1
0 Did Not Change 1
2
3 Changed for the Best

19 Description

19 Rating
-3 Changed for the Worst
-2
-1
0 Did Not Change 1
2
3 Changed for the Best

OBERST CAREGIVING BURDEN SCALE.docx

BCOS120715 - modified HF.doc

17.3 MRUQ-HF

MODIFIED RESOURCE UTILIZATION QUESTIONNAIRE FOR HEART FAILURE

(SOCIETAL PERSPECTIVE)

This is a series of questions about your health care that you have received during **past 3 months**.

1. During the **past 3 months**, how many times have you had **appointments or visits** at any of the following clinics or providers?

Clinics / Providers	Number of Appointments/Visits	How Long (hours)	Reason for Appointments/Visits
Cardiologist			
Home health services			

Nurse clinic visit			
Emergency department			
Hospitalization			
**Other healthcare appointments** Endocrinologist			
Nephrologist			
Ophthalmologist			
Allergist/Ear Nose Throat clinic			
Alternative/ homeopathic medicine			
Chiropractor			
Dentist			
Dermatologist			
Family physician/ internal medicine/ primary care physician			
Gastroenterologist			
Mental health professional			
Orthopedist			
Physical therapist			
Physician assistant			
Podiatrist			
Other:			

2. Not including the visits just listed, during the **past 3 months**, how many times did you communicate (**telephone**, **e-mail, text, fax**) with anyone (physician, nurse, or technical support) regarding your heart condition?

	Reason for contact	Number of contacts for information needed or communication	Type of communication (phone, e-mail, etc)
Physician			
Nurse practitioner			
Nurse educator			
Social worker			
Technical (e.g., pacemaker company)			

Other:			

3. Within the **past 3 months**, have you been visited in your home for **heart failure education**, attended a class, or a support/informational group? ❑Yes ❑No

3a. If **yes**, how long was each educational session and who participated in the education process with you.

	Purpose	Length of Session	How many times	Who participated with (spouse/child/ sibling/other)
Visited at home				
Attended a class				
Heart failure support group				
Other:				

4. Within the **past 3 months**, have you or other family members attended **mental health counseling** sessions for support? ❑Yes ❑No

4a. If **yes**, how long was each counseling session and who participated in the process with you.

	Purpose	Length of Session	Who participated with (spouse/child/sibling/other)
Session 1			
Session 2			
Session 3			
Session 4			

5. Approximately how much did you spend for **<u>purchase or rental of health care–related equipment/medications</u>** for your health care over the **past 3 months**?

Item related to **heart failure**	Where purchased (mail order, pharmacy, or clinics)	Cost that your insurance **did not** cover
Medication		
Scale for weighing self		
Oxygen		
Heart failure device		
Batteries for device		
Alcohol wipes		
Dressing tape		
Item related to **other conditions**	Where purchased (mail order, pharmacy, or clinics)	Cost that your insurance **did not** cover
Other medication		
Blood glucose meter		
Test strips		
Lancets		
Insulin (include all types)		
Insulin pen		
Insulin pump reservoirs and cartridges		
Insulin pump infusion sets		
Batteries for pump / meter		
Infusion set inserter		
Continuous glucose monitor		
Continuous glucose monitor sensor		
Ketosticks		
Supplies / food to treat hypoglycemia		
Pump accessories		
Medic alert bracelet or necklace		
Other supplies:		

Item related to **mobility**	Where purchased (mail order, pharmacy, or clinics)	Cost that your insurance **did not** cover
Wheelchair		
Scooter		
Walker		
Cane		
Hospital bed		
Ramp		
Shower chair		
commode		
Other: ________________		

6. Do you receive your health insurance from an employer? ❑Yes ❑No

6a. What is your **annual** health insurance premium cost? $___________

6b. What is your **annual** health insurance deductible? $___________

The following questions ask about the effect of your health problems on your ability to work and perform regular activities during **past 3 months**. Please fill in the blanks or circle a number, as indicated.

7. During the past 3 months, how much time (minutes) **each week** did you spend taking your medications, following your diet (making choices and eating), and weighing yourself? ___________minutes

8. During the past 3 months, how much time was spent taking care of technical issues (such as caring for heart related equipment)? ___________minutes

9. Are you currently employed (working for pay)? ❑Yes ❑No ❑Retired

► If you answered '**No' or 'Retired'**, skip Questions 10 ~ 13 and go to Question 14.

10. During the past 3 months, have you been absent from work because of illness?

❑Yes ❑No

10a. If **yes**, how many days? ___________, partial days: ___________

11. During the past 3 months, how many hours did you actually work? _______hours

12. Does your workplace employ a nurse? ❑Yes ❑No

12a. If **yes**, during the past 3 months, how much time (minutes) did you spend in the nurse's office during the day? ___________minutes

12b. How many days per week? ___________days

13. During the past 3 months, how much did your health problems affect your productivity *while you were working*?

(Think about days you were limited in the amount or kind of work you could do, days you accomplished less than you would like, or days you could not do your work as carefully as usual. If your health problems affected your work only a little, choose a low number. Choose a high number if health problems affected your work a great deal.)

Consider only how much *your heart failure* affected productivity *while you were working.* Circle a number.

Health problems had **no effect** on my work										Health problems **completely prevented** me from working
0	1	2	3	4	5	6	7	8	9	10

14. During the past 3 months, how much did your health problems affect your ability to do your regular daily activities, other than work at a job?

(By regular activities, we mean the usual activities you do, such as work around the house, shopping, child care, exercising, studying, and so forth. Think about times you were limited in the amount or kinds of activities you could do and times you accomplished less than you would like. If your health problems affected your activities only a little, choose a low number. Choose a high number if health problems affected your activities a great deal.)

Consider only how much *your heart failure* affected your ability to do your regular daily activities, other than work at a job. Circle a number.

Health problems had **no effect** on my daily activities										Health problems **completely prevented** me from doing my daily activities
0	1	2	3	4	5	6	7	8	9	10

18 LITERATURE REFERENCES

1. Adams KF, Jr., Fonarow GC, Emerman CL, et al. Characteristics and outcomes of patients hospitalized for heart failure in the United States: rationale, design, and preliminary observations from the first 100,000 cases in the Acute Decompensated Heart Failure National Registry (ADHERE). *Am Heart J.* 2005;149(2):209-216.

2. ADHERE Scientific Advisory Committee. *Acute Decompensated Heart Failure National Registry(ADHERE®) Core Module Q1 2006 Final Cumulative National Benchmark Report.* Scios, Inc; July 2006 2006.

3. Jencks SF, Williams MV, Coleman EA. Rehospitalizations among patients in the Medicare fee-for-service program. *The New England journal of medicine.* 2009;360(14):1418-1428.

4. Graff L, Orledge J, Radford MJ, Wang Y, Petrillo M, Maag R. Correlation of the Agency for Health Care Policy and Research congestive heart failure admission guideline with mortality: peer review organization voluntary hospital association initiative to decrease events (PROVIDE) for congestive heart failure. *Annals of emergency medicine.* 1999;34(4 Pt 1):429-437.

5. Pang PS, Jesse R, Collins SP, Maisel A. Patients with acute heart failure in the emergency department: do they all need to be admitted? *J Card Fail.* 2012;18(12):900-903.

6. Storrow AB, Jenkins CA, Self WH, et al. The burden of acute heart failure on U.S. emergency departments. *JACC Heart failure.* 2014;2(3):269-277.

7. Alexander P, Alkhawam L, Curry J, et al. Lack of evidence for intravenous vasodilators in ED patients with acute heart failure: a systematic review. *Am J Emerg Med.* 2014.

8. Collins SP, Levy PD, Lindsell CJ, et al. The rationale for an acute heart failure syndromes clinical trials network. *J Card Fail.* 2009;15(6):467-474.

9. Collins SP, Lindsell CJ, Pang PS, et al. Bayesian adaptive trial design in acute heart failure syndromes: Moving beyond the mega trial. *Am Heart J.* 2012;164(2):138-145.

10. Collins SP, Lindsell CJ, Storrow AB, et al. Early changes in clinical characteristics after emergency department therapy for acute heart failure syndromes: identifying patients who do not respond to standard therapy. *Heart Fail Rev.* 2012;17(3):387-394.

11. Collins SP, Storrow AB, Levy PD, et al. Early management of patients with acute heart failure: state of the art and future directions--a consensus document from the SAEM/HFSA acute heart failure working group. *Acad Emerg Med.* 2015;22(1):94-112.

12. Felker GM, Butler J, Collins SP, et al. Heart failure therapeutics on the basis of a biased ligand of the angiotensin-2 type 1 receptor. Rationale and design of the BLAST-AHF study (Biased Ligand of the Angiotensin Receptor Study in Acute Heart Failure). *JACC Heart failure.* 2015;3(3):193-201.

13. Heart Failure Executive C, Peacock WF, Fonarow GC, et al. Society of Chest Pain Centers Recommendations for the evaluation and management of the observation stay acute heart failure patient: a report from the Society of Chest Pain Centers Acute Heart Failure Committee. *Critical pathways in cardiology.* 2008;7(2):83-86.

14. Pang PS, Collins SP, Miro O, et al. The role of the emergency department in the management of acute heart failure: An international perspective on education and research. *Eur Heart J Acute Cardiovasc Care.* 2015.

15. Peacock WF, Braunwald E, Abraham W, et al. National Heart, Lung, and Blood Institute working group on emergency department management of acute heart failure: research challenges and opportunities. *J Am Coll Cardiol.* 2010;56(5):343-351.

16. Peacock WF, Fonarow GC, Ander DS, et al. Society of Chest Pain Centers recommendations for the evaluation and management of the observation stay acute heart failure patient-parts 1-6. *Acute cardiac care.* 2009;11(1):3-42.

17. Storrow AB, Lindsell CJ, Collins SP, et al. Standardized reporting criteria for studies evaluating suspected acute heart failure syndromes in the emergency department. *Journal of the American College of Cardiology.* 2012;60(9):822-832.

18. Weintraub NL, Collins SP, Pang PS, et al. Acute heart failure syndromes: emergency department presentation, treatment, and disposition: current approaches and future aims: a scientific statement from the American Heart Association. *Circulation.* 2010;122(19):1975-1996.

19. Collins SP, Pang PS, Butler J, Fonarow G, Metra M, Gheorghiade M. Revisiting cardiac injury during acute heart failure: further characterization and a possible target for therapy. *Am J Cardiol.* 2015;115(1):141-146.

20. Pang PS, Collins SP, Sauser K, et al. Assessment of dyspnea early in acute heart failure: patient characteristics and response differences between likert and visual analog scales. *Acad Emerg Med.* 2014;21(6):659-666.

21. Pang PS, Collins SP, Maisel A, Jesse R. Reply to: Decision algorithms are needed in acute heart failure in the emergency department. *J Card Fail.* 2013;19(2):147-148.

22. Collins SP, Pang PS, Fonarow GC, Yancy CW, Bonow RO, Gheorghiade M. Is hospital admission for heart failure really necessary?: the role of the emergency department and observation unit in preventing hospitalization and rehospitalization. *J Am Coll Cardiol.* 2013;61(2):121-126.

23. Pang PS, Collins SP, Storrow AB. Letter by Pang et al regarding article, "Early deaths in heart failure patients discharged from the emergency department: a population-based analysis". *Circulation Heart failure.* 2010;3(4):e22; author reply e23.

24. Mebazaa A, Pang PS, Tavares M, et al. The impact of early standard therapy on dyspnoea in patients with acute heart failure: the URGENT-dyspnoea study. *Eur Heart J.* 2010;31(7):832-841.

25. Collins SP, Pang PS, Lindsell CJ, et al. International variations in the clinical, diagnostic, and treatment characteristics of emergency department patients with acute heart failure syndromes. *Eur J Heart Fail.* 2010;12(11):1253-1260.

26. Pang PS, Tavares M, Collins SP, et al. Design and rationale of the URGENT Dyspnea study: an international, multicenter, prospective study. *American journal of therapeutics.* 2008;15(4):299-303.

27. Pang PS, Cleland JG, Teerlink JR, et al. A proposal to standardize dyspnoea measurement in clinical trials of acute heart failure syndromes: the need for a uniform approach. *Eur Heart J.* 2008;29(6):816-824.

28. Collins S, Storrow AB, Kirk JD, Pang PS, Diercks DB, Gheorghiade M. Beyond pulmonary edema: diagnostic, risk stratification, and treatment challenges of acute heart failure management in the emergency department. *Annals of emergency medicine.* 2008;51(1):45-57.

29. Pang PS, Levy P, Shah SJ. Treatment of acute heart failure in the emergency department. *Expert review of cardiovascular therapy.* 2013;11(9):1195-1209.

30. Mozaffarian D, Benjamin EJ, Go AS, et al. Heart Disease and Stroke Statistics-2016 Update: A Report From the American Heart Association. *Circulation.* 2015.

31. Heidenreich PA, Albert NM, Allen LA, et al. Forecasting the Impact of Heart Failure in the United States: A Policy Statement From the American Heart Association. *Circulation Heart failure.* 2013.

32. Maggioni AP, Dahlstrom U, Filippatos G, et al. EURObservational Research Programme: regional differences and 1-year follow-up results of the Heart Failure Pilot Survey (ESC-HF Pilot). *Eur J Heart Fail.* 2013;15(7):808-817.

33. Solomon SD, Dobson J, Pocock S, et al. Influence of nonfatal hospitalization for heart failure on subsequent mortality in patients with chronic heart failure. *Circulation.* 2007;116(13):1482-1487.

34. Setoguchi S, Stevenson LW, Schneeweiss S. Repeated hospitalizations predict mortality in the community population with heart failure. *Am Heart J.* 2007;154(2):260-266.

35. Krumholz HM. Post-hospital syndrome--an acquired, transient condition of generalized risk. *The New England journal of medicine.* 2013;368(2):100-102.

36. Hernandez AF, Greiner MA, Fonarow GC, et al. Relationship between early physician follow-up and 30-day readmission among Medicare beneficiaries hospitalized for heart failure. *JAMA : the journal of the American Medical Association.* 2010;303(17):1716-1722.

37. Rising KL, White LF, Fernandez WG, Boutwell AE. Emergency department visits after hospital discharge: a missing part of the equation. *Annals of emergency medicine.* 2013;62(2):145-150.

38. Vashi AA, Fox JP, Carr BG, et al. Use of hospital-based acute care among patients recently discharged from the hospital. *JAMA : the journal of the American Medical Association.* 2013;309(4):364-371.

39. Yancy CW, Jessup M, Bozkurt B, et al. 2013 ACCF/AHA guideline for the management of heart failure: a report of the American College of Cardiology Foundation/American Heart Association Task Force on Practice Guidelines. *J Am Coll Cardiol.* 2013;62(16):e147-239.

40. Collins SP, Peacock WF, Lindsell CJ, et al. S3 detection as a diagnostic and prognostic aid in emergency department patients with acute dyspnea. *Annals of emergency medicine.* 2009;53(6):748-757.

41. Yancy CW, Lopatin M, Stevenson LW, De Marco T, Fonarow GC. Clinical presentation, management, and in-hospital outcomes of patients admitted with acute decompensated heart failure with preserved systolic function: a report from the Acute Decompensated Heart Failure National Registry (ADHERE) Database. *J Am Coll Cardiol.* 2006;47(1):76-84.

42. Nieminen MS, Brutsaert D, Dickstein K, et al. EuroHeart Failure Survey II (EHFS II): a survey on hospitalized acute heart failure patients: description of population. *Eur Heart J.* 2006;27(22):2725-2736.

43. Buckley LF, Carter DM, Matta L, et al. Intravenous Diuretic Therapy for the Management of Heart Failure and Volume Overload in a Multidisciplinary Outpatient Unit. *JACC Heart failure.* 2016;4(1):1-8.

44. Institute of Medicine (U.S.). Committee on Quality of Health Care in America. *Crossing the quality chasm : a new health system for the 21st century.* Washington, D.C.: National Academy Press; 2001.

45. Storrow AB, Collins SP, Lyons MS, Wagoner LE, Gibler WB, Lindsell CJ. Emergency department observation of heart failure: preliminary analysis of safety and cost. *Congestive heart failure.* 2005;11(2):68-72.

46. Schrager J, Wheatley M, Georgiopoulou V, et al. Favorable bed utilization and readmission rates for emergency department observation unit heart failure patients. *Acad Emerg Med.* 2013;20(6):554-561.

47. Ponikowski P, Voors AA, Anker SD, et al. 2016 ESC Guidelines for the diagnosis and treatment of acute and chronic heart failure: The Task Force for the diagnosis and treatment of acute and chronic heart failure of the European Society of Cardiology (ESC)Developed with the special contribution of the Heart Failure Association (HFA) of the ESC. *Eur Heart J.* 2016;37(27):2129-2200.

48. Nohria A, Tsang SW, Fang JC, et al. Clinical assessment identifies hemodynamic profiles that predict outcomes in patients admitted with heart failure. *J Am Coll Cardiol.* 2003;41(10):1797-1804.

49. Dawson L, Zarin DA, Emanuel EJ, Friedman LM, Chaudhari B, Goodman SN. Considering usual medical care in clinical trial design. *PLoS Med.* 2009;6(9):e1000111.

50. Storrow A, Jenkins C, Self WH, et al. The Burden of Acute Heart Failure on US Emergency Departments. *Journal of The American College of Cardiology: Heart Failure.* 2014.

51. Teerlink JR, Cotter G, Davison BA, et al. Serelaxin, recombinant human relaxin-2, for treatment of acute heart failure (RELAX-AHF): a randomised, placebo-controlled trial. *Lancet.* 2013;381(9860):29-39.

52. O'Connor CM, Starling RC, Hernandez AF, et al. Effect of nesiritide in patients with acute decompensated heart failure. *The New England journal of medicine.* 2011;365(1):32-43.

53. Massie BM, O'Connor CM, Metra M, et al. Rolofylline, an adenosine A1-receptor antagonist, in acute heart failure. *The New England journal of medicine.* 2010;363(15):1419-1428.

54. Joosten YA, Israel TL, Williams NA, et al. Community Engagement Studios: A Structured Approach to Obtaining Meaningful Input From Stakeholders to Inform Research. *Acad Med.* 2015;90(12):1646-1650.

55. Ariti CA, Cleland JG, Pocock SJ, et al. Days alive and out of hospital and the patient journey in patients with heart failure: Insights from the candesartan in heart failure: assessment of reduction in mortality and morbidity (CHARM) program. *Am Heart J.* 2011;162(5):900-906.

56. Allen LA, Hernandez AF, O'Connor CM, Felker GM. End points for clinical trials in acute heart failure syndromes. *J Am Coll Cardiol.* 2009;53(24):2248-2258.

57. Joynt KE, Jha AK. Thirty-day readmissions--truth and consequences. *The New England journal of medicine.* 2012;366(15):1366-1369.

58. Stevenson LW, Hellkamp AS, Leier CV, et al. Changing preferences for survival after hospitalization with advanced heart failure. *J Am Coll Cardiol.* 2008;52(21):1702-1708.

59. Sauser K, Spertus JA, Pierchala L, Davis E, Pang PS. Quality of life assessment for acute heart failure patients from emergency department presentation through 30 days after discharge: a pilot study with the Kansas City Cardiomyopathy Questionnaire. *J Card Fail.* 2014;20(1):18-22.

60. Green CP, Porter CB, Bresnahan DR, Spertus JA. Development and evaluation of the Kansas City Cardiomyopathy Questionnaire: a new health status measure for heart failure. *J Am Coll Cardiol.* 2000;35(5):1245-1255.

61. Heidenreich PA, Spertus JA, Jones PG, et al. Health status identifies heart failure outpatients at risk for hospitalization or death. *Journal of the American College of Cardiology.* 2006;47(4):752-756.

62. Spertus J, Peterson E, Conard MW, et al. Monitoring clinical changes in patients with heart failure: a comparison of methods. *Am Heart J.* 2005;150(4):707-715.

63. Albert NM, Yancy CW, Liang L, et al. Use of aldosterone antagonists in heart failure. *JAMA : the journal of the American Medical Association.* 2009;302(15):1658-1665.

64. Hernandez AF, Fonarow GC, Liang L, Heidenreich PA, Yancy C, Peterson ED. The need for multiple measures of hospital quality: results from the Get with the Guidelines-Heart Failure Registry of the American Heart Association. *Circulation.* 2011;124(6):712-719.

65. Heidenreich PA, Hernandez AF, Yancy CW, Liang L, Peterson ED, Fonarow GC. Get With The Guidelines program participation, process of care, and outcome for Medicare patients hospitalized with heart failure. *Circ Cardiovasc Qual Outcomes.* 2012;5(1):37-43.

66. Bonow RO, Ganiats TG, Beam CT, et al. ACCF/AHA/AMA-PCPI 2011 Performance Measures for Adults With Heart Failure: A Report of the American College of Cardiology Foundation/American Heart Association Task Force on Performance Measures and the American Medical Association-Physician Consortium for Performance Improvement. *J Am Coll Cardiol.* 2012.

67. Yancy CW, Jessup M, Bozkurt B, et al. 2016 ACC/AHA/HFSA Focused Update on New Pharmacological Therapy for Heart Failure: An Update of the 2013 ACCF/AHA Guideline for the Management of Heart Failure: A Report of the American College of Cardiology/American Heart Association Task Force on Clinical Practice Guidelines and the Heart Failure Society of America. *Circulation.* 2016.

68. Humphrey L, Kulich K, Deschaseaux C, Blackburn S, Maguire L, Stromberg A. The Caregiver Burden Questionnaire for Heart Failure (CBQ-HF): face and content validity. *Health and quality of life outcomes.* 2013;11:84.

69. Krumholz HM, Butler J, Miller J, et al. Prognostic importance of emotional support for elderly patients hospitalized with heart failure. *Circulation.* 1998;97(10):958-964.

70. Jung M, Titler M, Riley P, Bleske B, Pressler SJ. A New Measure of Healthcare Resource Utilization in Heart Failure: Development and Content Validity Evaluation. *Journal of Cardiac Failure.* 2015;8(21):s116.

71. Smaldone A, Tsimicalis A, Stone PW. Measuring resource utilization in patient-oriented comparative effectiveness research: a psychometric study of the Resource Utilization Questionnaire. *Research and theory for nursing practice.* 2011;25(2):80-106.

72. Austin PC. Balance diagnostics for comparing the distribution of baseline covariates between treatment groups in propensity-score matched samples. *Statistics in medicine.* 2009;28(25):3083-3107.

73. Spertus JA, Jones PG. Development and Validation of a Short Version of the Kansas City Cardiomyopathy Questionnaire. *Circ Cardiovasc Qual Outcomes.* 2015;8(5):469-476.

74. Eapen ZJ, Liang L, Fonarow GC, et al. Validated, electronic health record deployable prediction models for assessing patient risk of 30-day rehospitalization and mortality in older heart failure patients. *JACC Heart failure.* 2013;1(3):245-251.

75. Shen C, Li X, Li L. Inverse probability weighting for covariate adjustment in randomized studies. *Statistics in medicine.* 2014;33(4):555-568.
