# Supplementary material for: Short-Stay Units vs Routine Admission From the Emergency Department in Patients With Acute Heart Failure: The SSU-AHF Randomized Clinical Trial
Source: JAMA Netw Open. 2024 Jan 10;7(1):e2350511. doi: 10.1001/jamanetworkopen.2023.50511 (PMC10782263; doi:10.1001/jamanetworkopen.2023.50511)
Supplement: Supplement 2. — eTable 1. Eligibility Criteria eTable 2. Study Sites eTable 3. Study Outcomes eTable 4. KCCQ Power Calculations eTable 5. Comparison of Treatment Groups With Both Discharge and 30-Day KCCQ eTable 6. ED AHF Medications eTable 7. Day of Discharge Medications eTable 8. 90-Day Secondary Outcomes eTable 9. SSU Failures Compared With Hospitalization Study Group [file jamanetwopen-e2350511-s002.pdf]

Supplemental Online Content

Pang PS, Berger DA, Mahler SA, et al. Short-stay units vs routine admission from the emergency department in patients with acute heart failure: the SSU-AHF randomized clinical trial. *JAMA Netw Open*. 2024;7(1):e2350511. doi:10.1001/jamanetworkopen.2023.50511

eTable 1. Eligibility Criteria

eTable 2. Study Sites

eTable 3. Study Outcomes

eTable 4. KCCQ Power Calculations

eTable 5. Comparison of Treatment Groups With Both Discharge and 30-Day KCCQ

eTable 6. ED AHF Medications

eTable 7. Day of Discharge Medications

eTable 8. 90-Day Secondary Outcomes

eTable 9. SSU Failures Compared With Hospitalization Study Group

This supplemental material has been provided by the authors to give readers additional information about their work.

© 2024 Pang PS et al. *JAMA Network Open.*

eTable 1. Eligibility Criteria

Inclusion:

1) ED physician clinical diagnosis of AHF
2) Planned admission for AHF
3) Systolic blood pressure > 100mmHg[a], heart rate < 115bpm. The last HR and SBP should be within 1 hour of randomization.
4) Previous history of HF

Exclusion Criteria

1) Exclusion: Patients hospitalized within the last 30 days ONLY if the institution mandates these patients are observed. Otherwise, these patients remained eligible.
2) Transplanted organ of any kind or ventricular assist device in place;
3) End stage renal disease, on dialysis, or eGFR < 20 mL/min/1.73m2;
4) Acute coronary syndrome (e.g. EKG changes consistent with ischemia or troponin elevation secondary to ACS as per the treating ED clinician; patients with an isolated elevated troponin were eligible);
5) Other acute co-morbid conditions (e.g. sepsis, altered mental status);
6) Patients who require ventilatory support of any kind or intravenous vasodilators/vasopressor/inotropic support at the time of ED disposition
7) Pregnant patients or any patient who has been pregnant in the last 3 months
8) ≤ 18 years of age
9) Any patient who in the opinion of the clinician or investigator should not be in an obs unit or requires ICU level care or will require inpatient rehabilitation or skilled nursing facility after discharge from the ED or hospital
10) Planned discharge from the emergency department
11) De novo (new onset) AHF

a - Patients with atrial fibrillation but controlled HR were eligible

© 2024 Pang PS et al. *JAMA Network Open.*

eTable 2. Study Sites

**Site and Location**	Primary Investigator	Coordinator(s)
**Indiana University, Indiana**	Peter Pang, MD	Mette Cole MD and Morgan White
**Allegheny Health Network, Pennsylvania**	Arvind Venkat, MD	Kelly Szabo
**Ohio State, Ohio**	Jason Bischof MD	Michael Hill
**University of Alabama, Birmingham**	Erik Hess	Joel Rodgers
**Vanderbilt, Tennessee**	Sean Collins	Shannon Pugh
**Wake Forest, North Carolina**	Simon Mahler	Stephanie Elliot
**Wayne State University, Michigan**	Philip Levy, MD	Sarah Meram
**William Beaumont, Michigan**	David Berger, MD	Mara Branoff
**Wash U, Missouri**	Douglas Char, MD	Stacey House, MD
**U of Mississippi**	Alan Jones, MD	Rebekah Peacock
**Henry Ford Hospital, Michigan**	Joseph Miller, MD	Julian Suszanski
**UT Southwestern, Texas**	Deb Diercks, MD	Kimberly Zepeda

© 2024 Pang PS et al. *JAMA Network Open.*

eTable 3. Study Outcomes

Original Study Outcomes:
Primary Outcome: Days alive and out of hospital (DAOOH) at 30-days post-randomization.

Secondary Outcomes:

1) Quality of life as measured by Kansas City Cardiomyopathy Questionnaire
2) Cost-effectiveness analysis between the two arms at 30-days

Exploratory Outcomes[a]:

1) Caregiver burden as measured by two Caregiver Burden Scales at 30-days;
2) Cost-Effectiveness of the SSU AHF strategy of care at 90-days;
3) Resource utilization measured by the Modified Resource Utilization Questionnaire for Heart Failure (mRUQ-HF) at 90-days
4) All-cause mortality and re-hospitalization at 30 and 90 days
5) Days alive and out of hospital at 90-days
6) HF Guideline adherence at time of discharge

Revised Study Outcomes:

Primary Outcome: Quality of Life as measured by KCCQ-12 at 30-days

Secondary Outcomes:

1) 30 and 90-day DAOOH post-randomization and post-discharge,
2) Quality of Life as measured by the SF-12 at 30-days post-randomization,
3) All-cause mortality and re-hospitalization at 30 and 90-days

a- Cost effectiveness is not reported nor mRUQ due to truncation of study enrollment

© 2024 Pang PS et al. *JAMA Network Open.*

eTable 4. KCCQ Power Calculations

Below are detectable differences in KCCQ change between the two treatment modalities corresponding to various hypothetical patient attrition with 80% power and two-sided type I error 0.05, assuming data missing completely at random.

Sample size (both arms combined)	% of data attrition	Difference in KCCQ change
480	10%	5.3
360	33%	6.2
240	55%	7.6
120	78%	10.7

© 2024 Pang PS et al. *JAMA Network Open.*

eTable 5. Comparison of Treatment Groups with both Discharge and 30 day KCCQ

	SSU		Hospitalization		
Variable name	*N*	*Statistics*	*N*	*Statistics*	*P-value*
Age (years), mean (sd)	64	65.5 (13.8)	67	67.7 (15.2)	0.39
Age ≥ 75 years, n (%)	64	16 (25.0%)	67	24 (35.8%)	0.19
Male Gender, n (%)	64	44 (68.8%)	67	36 (53.7%)	0.11
Race, n (%)	64		67		0.66
Black		33 (51.6%)		36 (53.7%)	
White		28 (43.8%)		30 (44.8%)	
Other		3 (4.7%)		1 (1.5%)	
Hispanic or Latino?, n (%)	64	1 (1.6%)	66	0 (0.0%)	0.49
Any ED Visit or hospitalization in the past 6 months, n (%)	64	46 (71.9%)	67	45 (67.2%)	0.58
CAD, n (%)	64	36 (56.3%)	67	35 (52.2%)	0.73
Hypertension (HTN), n (%)	64	55 (85.9%)	67	61 (91.0%)	0.42
Diabetes, n (%)	64	37 (57.8%)	67	33 (49.3%)	0.38
Chronic Kidney Disease, n (%)	64	25 (39.1%)	67	26 (38.8%)	1.00
On dialysis, n (%)	25	0 (0.0%)	26	0 (0.0%)	
Depression history, n (%)	64	15 (23.4%)	67	17 (25.4%)	0.84
Asthma or COPD, n (%)	64	21 (32.8%)	67	20 (29.9%)	0.85
Pacemaker, n (%)	64	16 (25.0%)	67	8 (11.9%)	0.07
ICD, n (%)	64	15 (23.4%)	66	17 (25.8%)	0.84
Atrial fibrillation or Atrial flutter, n (%)	64	27 (42.2%)	67	33 (49.3%)	0.48
CVA/TIA, n (%)	64	15 (23.4%)	67	16 (23.9%)	1.00
Heart rate, mean (sd)	64	80.6 (13.8)	67	83.4 (17.3)	0.30
Respiratory rate, mean (sd)	64	20.0 (4.4)	67	19.7 (2.8)	0.72
Systolic blood pressure, mean (sd)	64	141.0 (24.5)	67	140.4 (28.8)	0.89
Diastolic blood pressure, mean (sd)	64	82.3 (19.0)	67	81.0 (19.2)	0.69
Oxygen saturation, mean (sd)	64	97.3 (2.6)	67	97.2 (2.3)	0.89
BMI (kg/m2) - measured weight, mean (sd)	22	36.3 (12.3)	25	35.7 (12.0)	0.87
Sodium value, median (IQR)	64	139.0 (137.0 - 141.0)	67	139.0 (137.0 - 141.0)	0.65
Creatinine value, median (IQR)	64	1.3 (1.0 - 1.6)	67	1.2 (0.9 - 1.4)	0.54
BNP value, median (IQR)	61	758.0 (414.0 - 1587.0)	64	663.5 (359.5 - 1792.0)	0.39

© 2024 Pang PS et al. *JAMA Network Open.*

	SSU		Hospitalization		
Variable name	*N*	*Statistics*	*N*	*Statistics*	*P-value*
Troponin-I value:, median (IQR)	62	0.0 (0.0 - 0.1)	59	0.0 (0.0 - 0.1)	0.20
LV Ejection Fraction, mean (sd)	34	35.6 (16.6)	43	41.5 (13.6)	0.09
Left Ventricular Ejection Fraction, n (%)	34		43		0.03
LVEF <40		23 (67.6%)		17 (39.5%)	
LVEF 40-55		6 (17.6%)		19 (44.2%)	
LVEF >55		5 (14.7%)		7 (16.3%)	
KCCQ-12 Score, mean (sd)	64	36.0 (20.6)	67	30.9 (21.7)	0.17

© 2024 Pang PS et al. *JAMA Network Open.*

eTable 6. ED AHF Medications

	SSU		*Hospitalization*		
	N	*Statistics*	*N*	*Statistics*	*p-value*
Lasix/furosemide, n (%)	93	87 (93.5%)	100	87 (87.0%)	0.15
Number of IV Lasix doses in ED, n (%)	83		85		0.84
1		68 (81.9%)		71 (83.5%)	
2		15 (18.1%)		14 (16.5%)	
Total IV Dose (mg) of Lasix, mean (sd)	82	61.0 (30.4)	85	55.5 (32.0)	0.26
Total PO Dose (mg) of Lasix, mean (sd)	8	45.0 (17.7)	3	73.3 (30.6)	0.08
Nitroglycerine, n (%)	93	12 (12.9%)	100	18 (18.0%)	0.43
Nitroglycerine - IV, n (%)	12	0 (0.0%)	18	2 (11.1%)	0.50
Nitroglycerine IV Bolus, n (%)	12	2 (16.7%)	18	0 (0.0%)	0.15
Nitroprusside, n (%)	91	1 (1.1%)	98	1 (1.0%)	1.00
Noninvasive Ventilation, n (%)	93	6 (6.5%)	100	4 (4.0%)	0.53
Inotropes/Vasopressors, n (%)	93	0 (0.0%)	100	0 (0.0%)	n/a

© 2024 Pang PS et al. *JAMA Network Open.*

eTable 7. Day of Discharge Medications

	SSU-Fail		SSU-no fail		Hospitalization		
	N	Statistics	N	Statistics	N	Statistics	P-value
Angiotensin Receptor Blockers, n (%)	39	7 (17.9%)	54	12 (22.2%)	98	14 (14.3%)	0.46
ACE-Inhibitors, n (%)	39	11 (28.2%)	54	10 (18.5%)	98	25 (25.5%)	0.48
ARNI, n (%)	39	3 (7.7%)	54	3 (5.6%)	98	6 (6.1%)	0.86
Aspirin, n (%)	39	21 (53.8%)	54	26 (48.1%)	98	53 (54.1%)	0.76
other antiplatelet (ticagrelor/clopidogrel, prasugrel), n (%)	39	5 (12.8%)	54	13 (24.1%)	98	12 (12.2%)	0.14
Albuterol, n (%)	39	5 (12.8%)	54	9 (16.7%)	98	14 (14.3%)	0.89
Beta-Blockers, n (%)	39	25 (64.1%)	54	39 (72.2%)	98	68 (69.4%)	0.70
Isosorbide Dinitrate/Hydralazine, n (%)	39	5 (12.8%)	54	9 (16.7%)	98	19 (19.4%)	0.67
Calcium channel blockers, n (%)	39	4 (10.3%)	54	10 (18.5%)	98	22 (22.4%)	0.28
Anticoagulation, n (%)	39	23 (59.0%)	54	15 (27.8%)	98	52 (53.1%)	0.003
Digoxin, lanoxin or digitalis, n (%)	39	1 (2.6%)	54	0 (0.0%)	98	1 (1.0%)	0.45
Furosemide, n (%)	39	18 (46.2%)	54	37 (68.5%)	98	58 (59.2%)	0.10
Torsemide, n (%)	39	7 (17.9%)	54	3 (5.6%)	98	12 (12.2%)	0.18
Bumetanide, n (%)	39	3 (7.7%)	54	2 (3.7%)	98	5 (5.1%)	0.76
Metolazone, n (%)	39	2 (5.1%)	54	0 (0.0%)	98	1 (1.0%)	0.18
HCTZ/Hydrochlorothiazide, n (%)	39	0 (0.0%)	54	1 (1.9%)	98	1 (1.0%)	1.00
Chlorthalidone, n (%)	39	0 (0.0%)	54	0 (0.0%)	98	1 (1.0%)	1.00
Nitroglycerine and other nitrates, n (%)	39	4 (10.3%)	54	4 (7.4%)	98	10 (10.2%)	0.85
Mineralocorticoid Receptor Antagonist, n (%)	39	8 (20.5%)	54	10 (18.5%)	98	23 (23.5%)	0.80

© 2024 Pang PS et al. *JAMA Network Open.*

eTable 8. 90-Day Secondary Outcomes

	SSU		*Usual Care*		
	N	*Statistics*	*N*	*Statistics*	*P-value*
90-day follow-up, n (%)	93	82 (88.2%)	98	90 (91.8%)	0.47
SF-12 Physical Component Scale, mean (sd)	61	38.8 (8.8)	58	37.4 (8.7)	0.40
SF-12 Mental Component Scale, mean (sd)	61	38.6 (7.4)	58	39.7 (6.8)	0.41
KCCQ-12 Score, mean (sd)	63	56.6 (30.1)	58	53.2 (25.7)	0.51
90-day All-cause death or re-hospitalization[a]	77	36 (46.8%)	85	35 (41.2%)	0.66[b]
Unscheduled E.D. visit during 90 days post-original hospital discharge, n (%)	82	44 (53.7%)	90	38 (42.2%)	0.17
Unscheduled E.D. visit during 90 days post-randomization, n (%)	82	41 (50.0%)	90	38 (42.2%)	0.36
Unscheduled E.D. visit for Heart Failure during 90 days post-randomization, n (%)	82	26 (31.7%)	90	24 (26.7%)	0.50
Unscheduled hospital admission during 90 days post-randomization, n (%)	82	35 (42.7%)	90	32 (35.6%)	0.35
Unscheduled hospital admission for Heart Failure during 90 days post-randomization, n (%)	82	26 (31.7%)	90	21 (23.3%)	0.23

a- Only in subjects with follow-up

b- Log-rank and stratified log-rank p-values are testing time from randomization to the earlier of all-cause death or rehospitalization (days)

© 2024 Pang PS et al. *JAMA Network Open.*

eTable 9. SSU Failures Compared With Hospitalization Study Group

	SSU Fails		*Hospitalization*		
	N	*Statistics*	*N*	*Statistics*	*P-value*
Minutes from Initial Presentation to Randomization, mean (sd)	39	233.5 (130.1)	100	230.8 (186.8)	0.92
Outcomes					
Days in ED/Hospital since Randomization, median (IQR)	39	4.0 (2.8 – 10.1)	100	3.1 (2.0 – 5.9)	0.03
KCCQ-12 Score (discharge), mean (sd)	38	31.7 (19.3)	97	33.1 (23.1)	0.74
KCCQ-12 Score (30-Days), mean (sd)	29	50.3 (24.0)	68	45.8 (23.8)	0.39
Number of days in ED/Hospital during 30 days after hospital discharge, mean (sd)	37	0.4 (1.5)	93	1.2 (2.6)	0.04
Number of days in ED/Hospital during 30 days post-randomization including initial presentation, mean (sd)	37	7.6 (6.5)	93	5.6 (4.8)	0.09
30 Days DAOOH -based upon original hospital discharge, mean (sd)	37	30.0 (27.0-30.0)	93	30.0 (27.4 – 30.0)	0.62
30 Days DAOOH -from randomization, mean (sd)	37	25.2 (18.9-27.2)	95	25.4 (22.0 – 27.7)	0.24
Medications Given during ED Phase of Management					
Lasix/furosemide given, n (%)	39	35 (89.7%)	100	87 (87.0%)	0.78
Number of PO Lasix doses in ED, n (%)	3		3		1.00
1		3 (100.0%)		2 (66.7%)	
2		0 (0.0%)		1 (33.3%)	
Number of IV Lasix doses in ED, n (%)	34		85		0.30
1		25 (73.5%)		71 (83.5%)	
2		9 (26.5%)		14 (16.5%)	
Total IV Dose (mg) of Lasix, mean (sd)	33	68.8 (30.8)	85	55.5 (32.0)	0.04
Total PO Dose (mg) of Lasix, mean (sd)	3	53.3 (23.1)	3	73.3 (30.6)	0.42
Nitroglycerine, n (%)	39	6 (15.4%)	100	18 (18.0%)	0.81
Noninvasive Ventilation, n (%)	39	4 (10.3%)	100	4 (4.0%)	0.22
Inotropes/Vasopressors, n (%)	39	0 (0.0%)	100	0 (0.0%)	n/a

© 2024 Pang PS et al. *JAMA Network Open.*

© 2024 Pang PS et al. *JAMA Network Open.*
