# Supplementary material for: Short-Stay Units vs Routine Admission From the Emergency Department in Patients With Acute Heart Failure: The SSU-AHF Randomized Clinical Trial
Source: JAMA Netw Open. 2024 Jan 10;7(1):e2350511. doi: 10.1001/jamanetworkopen.2023.50511 (PMC10782263; doi:10.1001/jamanetworkopen.2023.50511)
Supplement: Supplement 3. — Data Sharing Statement [file jamanetwopen-e2350511-s003.pdf]

# Data Sharing Statement

Pang. Short-Stay Units vs Routine Admission From the Emergency Department in Patients With Acute Heart Failure. *JAMA Netw Open*. Published January 10, 2024. doi:10.1001/jamanetworkopen.2023.50511

**Data**
**Data available:** Yes
**Data types:** Participant data with identifiers
**How to access data:** We will create new random identification numbers without site identifiers to replace the original identification numbers,m per our study protocol. ppang@iu.edu
**When available:** With publication

**Supporting Documents**
**Document types:** None

**Additional Information**
**Who can access the data:** Researchers whose proposed use of the data has been approved
**Types of analyses:** For any purpose to advance medicine
**Mechanisms of data availability:** After approval of a proposal
**Any additional restrictions:** A signed data access agreement or datatransfer agreement may be necessary
